# Knowing the ABCs: teaching the principles of radiology to medical students in Turkey

**DOI:** 10.1186/s12909-022-03885-8

**Published:** 2022-12-12

**Authors:** Emre Altinmakas, Omer Faruk Dogru, Umut Yucel, Görkem Ayas, Ayşe Sena Balcı, Munevver Duran, Hakan Doğan, Serageldin Kamel, Parth Patel, Khaled M Elsayes

**Affiliations:** 1 Department of Radiology, Koç University School of Medicine, 34010 Istanbul, Turkey; 2 Department of Diagnostic, Molecular and Interventional Radiology, Icahn School of Medicine at Mount Sinai, New York, NY USA; 3 Medical School, Istanbul Medipol University International School of Medicine, Istanbul, Turkey; 4 Medical School, Bahcesehir University School of Medicine, Istanbul, Turkey; 5 Medical School, Koç Üniversitesi School of Medicine, İstanbul, Turkey; 6 Medical School, Istanbul University Cerrahpasa School of Medicine, Istanbul, Turkey; 7 The University of Texas Southwestern Medical Center, Dallas, TX USA; 8 University of Texas Medical Branch School of Medicine, Galveston, TX USA; 9 Department of Lymphoma and Myeloma, University of Texas MD Anderson Cancer Center, Houston, TX USA; 10 Department of Abdominal Imaging, University of Texas MD Anderson Cancer Center, Houston, TX USA

**Keywords:** Medical students, Radiology, Education

## Abstract

**Background:**

Radiology education in Turkey is mainly taught during clinical years of medical school and often lacks main principles. Exposure to the fundamentals of radiology at an early stage of medical education may drastically help students generate a better understanding of radiology and expand their interest in the specialty. With the Principles of Radiology Course that we provided, pre- and post-session tests, and assessment survey at the end of the course, we aimed to evaluate the effectiveness of such an online course among Turkish medical students.

**Methods:**

A total of nine online sessions on imaging modalities principles was developed by radiology professors. Each session was given through Zoom by radiologists from different U.S.-institutions to Turkish medical students from state (*n* = 33) and private (*n* = 8) universities. Pretests and post-tests were given to participants via Qualtrics before and after each session, respectively. Paired two-sample t-tests were conducted to detect the variance and *p*=-.05 was used as the significance level. An evaluation survey was distributed at the end of the course to collect their feedback through SurveyMonkey.

**Results:**

A total of 1,438 predominantly Turkish (99.32%) medical students engaged with this course. An average of 506 students completed both pre-test and post-test. There was a statistically significant (*p* < .001) increase in the scores in post-test (mean[range]:7.58[5.21–8.53]) relative to pre-test (mean[range]:5.10[3.52–8.53]). Four hundred and thirty-nine participants (F/M:63.33%/35.54%) completed the end-of-course survey. A total of 71% and 69.70% of the participants *strongly agreed* that the course would be useful in their clinical practice and had increased their understanding of radiology. They also reported that their level of confidence in the subjects had increased 68% and reached a weighted average of 3.09/4. The survey revealed that 396 (90.21%) of the participants *strongly* or *somewhat agree* that introductory principles and concepts should be presented in earlier years of medical education. Compared to in-person education, 358 (81.55%) found the course *extremely* or *very convenient.*

**Conclusion:**

Online lecture series consisting of the principles of the radiological imaging modalities can be offered to Turkish medical students to enhance their grasp of the various imaging modalities and their correct clinical application.

**Supplementary Information:**

The online version contains supplementary material available at 10.1186/s12909-022-03885-8.

## Background

The demand for quality radiology education as part of the medical curriculum has never been greater, as it allows for the visualization and conceptualization of intangible elements of human disease and brings into clinical practice the theoretical knowledge attained through anatomy, physiology, and pathology lectures. Gaining a strong foundation of core radiology knowledge is critical for medical students, as they will need these skills in their future clinical practices regardless of their specialty [1]. Exposing students to radiology in the early stages of their medical school education has been shown to help them make informed decisions when choosing a specialty [2–4].

In Turkey, like most other European countries, there is no standardized approach to the scheduling of educational programs or the amount of undergraduate radiology education content in medical schools [5, 6]. Despite several frameworks proposed by the European Society of Radiology (ESR), medical schools continue to develop their approaches to medical education, and significant variations exist, sometimes within the same nation [7, 8]. In a survey reported by the ESR, radiology education was found to be underrepresented in the current undergraduate curriculum when its role in medicine was taken into account [9]. Most radiology education content is delivered in the 4th and 5th years of medical education rather than in the pre-clinical years [9]. According to the same survey, radiology is also mostly taught in modules that are integrated with other clinical sciences, making it less pragmatically useful for students to study the topics for their exams and providing an inducement for students to skip radiological topics such as radiological anatomy [9].

The COVID-19 pandemic has drastically affected our approach to education worldwide. In March 2020, 61 countries began implementing measures to close down schools and universities [10]. The shutdown of classrooms and lecture halls has resulted in a surge of investment and interest in online education and distance learning, with a focus on developing new teaching strategies [11]. Medical schools have also been following this trend; with many engaging in online curricula, students have spent almost two full academic years with restricted access to their clinical rotations and patient interactions [12]. In terms of radiology education, similar steps had been taken before the pandemic, with more time allocated to case-based learning, teaching in small groups, hands-on workshops, and trainees going into reading rooms to learn more about radiological images; all of which have been particularly diminished during the pandemic [13].

To accommodate the conditions brought upon us by the pandemic, foster international collaborations in improving the quality of undergraduate medical education, and abide by the ESR guidelines on how radiological curriculum could be improved, we have designed, implemented, and evaluated a series of online courses in Turkey called the “Principles of Radiology”. Our goal was to evaluate the effectiveness of such an online course among Turkish medical students. To the best of our knowledge, this is the first article assessing, from the students’ point of view, radiology education in Turkish medical schools with an online radiology course completed nationwide.

## Methods

This observational study was approved by Baylor College of Medicine institutional review board (IRB) (protocol number: H-47,848). A written informed consent was obtained from each participant upon their application to the seminar.

### Course description

An online, free of charge multidisciplinary diagnostic radiology education course was designed and implemented by radiology educators from different United States based institutions as an addendum to the ongoing Turkish radiology curriculum. The course director recruited instructors from different institutions with expertise in the discussed topics and teaching to medical students. A similar design had been conducted among medical students in the United States, Canada, and Egypt before. The goal was to teach the principles of commonly used imaging modalities with example clinical applications. The final selected imaging modalities and topics are listed in Table 1. The course content and learning objectives include all of the obligatory learning objectives of “the Turkish National Core Education Programme”. The course, also, involved some of the recommended but not obligatory learning objectives. The course language was English. The reason for this language was that both the lecturers were from the USA and all the medical schools in Turkey provide an English preparatory-year or preparatory-class. A total of 9 sessions were held over 9 weeks. Each session lasted on average for 90 min and usually included a 60-minute didactic lecture on the concepts, history, and evolution of each imaging modality followed by examples of clinical scenarios. A pre-test and post-tests were administered at the beginning and end of the lecture, respectively and fifteen minutes on average were dedicated to questions and answers.

**Table 1:** Pre-test and post-test scores for each session

Session	No. of Students Completing Both Tests	Pre-test Score Mean (SD)	Post-test Score Mean SD)	% Change	*P* Value
X-Ray	704	4.51 (1.63)	7.89 (1.95)	74.94	< .001
Ultrasonography	469	6.13 (1.92)	8.23 (1.90)	34.26	< .001
Computed Tomography	521	5.25 (2.31)	7.89 (1.82)	50.29	< .001
Magnetic Resonance Imaging	496	4.33 (1.92)	7.96 (2.15)	83.83	< .001
Positron Emission Tomography	442	5.21 (1.91)	7.59 (1.68)	45.68	< .001
Contrast Agents	468	5.44 (1.60)	6.86 (1.71)	26.10	< .001
Roentgen to Artificial Intelligence	460	3.52 (1.64)	5.21 (1.96)	48.01	< .001
Interventional Radiology	436	6.68 (2.67)	8.53 (2.01)	27.69	< .001
Chest Radiographs	560	4.87 (2.16)	8.06 (2.12)	65.50	< .001

### Online learning platform

The course sessions were delivered via Zoom Webinar (Zoom Video Communications, San Jose, CA, zoom.us), a video conferencing platform which accommodates large numbers of attendees. It also provides several features that help moderating the sessions and organizing the discussions. These features include a chat box, a question-and-answer session organizer, and added controls of who can share screen, audio, and video. A built-in polling feature was also utilized during several sessions to encourage participation and provide the speaker with information about the students’ level of knowledge about the session’s topic.

### Student enrollment

In order to have a cohort that was representative of Turkish medical students, efforts were made to communicate with and assign student ambassadors in multiple medical schools in Turkey including state and private ones. The course was advertised through the student ambassadors using different channels such as social media platforms, emails, and among interest groups. An electronic registration form was distributed and interested students were asked to sign-up.

### Tests and test score analysis

Before and after each session, attendees were required to take a pre-test and a post-test, respectively, consisting of 10–15 multiple choice questions via Qualtrics (Qualtrics, Provo, UT). The pre-tests covering the main learning objectives measured the students’ knowledge of the topic to be presented in that session, and the same questions were asked again at the end of the session in the post-test. A handout was also distributed with each lecture to summarize the essential material addressed in that lecture. For each session, a different student ambassador was in charge of arranging contact with the panelists in order to develop questions for the tests and information for the handout. Each participant was given an individual anonymous code, which was used to pair their pre-test and post-test scores. A paired two-sample *t*-test was used to compare the means between pre-test and post-test scores of each session where *P* < .05 was considered to be significant.

### Course final evaluation survey

A course assessment survey was distributed to attendees using SurveyMonkey at the end of the course. It included 35 questions on student demographics, existing radiology education at their institution, perceived efficacy of the course in comparison to the existing radiology education, usefulness of the course for future practice, and overall enthusiasm about radiology. The survey also included questions about the relevance of session topics, each session’s rating, the usefulness of the post-session handouts, and instructor quality. All test and survey data were collected and graphed for analysis.

## Results

Based on the president of Council of higher Education, there are a total of 110,331 medical students in Turkey: of which 92,159 studying in public universities and 18,152 studying in foundation universities (https://www.aa.com.tr/tr/egitim/yok-baskani-ozvar-turkiyede-110-bin-331-ogrenci-tip-fakultelerinde-okuyor/2458569). From 41 different universities, a total of 1,458 medical students registered for this online radiology course. Of those, 1,256 (86.16%) students were from 33 different state universities, and 202 (13.85%) students were from eight different private (non-profit foundations) universities. An average of 506 medical students completed both the pre-test and the post-test for each of the sessions. Mean pre-test and post-test scores were 5.10/10 (range, 3.52–6.68) and 7.58/10 (range, 5.21–8.53), respectively. For each session, a statistically significant (*p* < .001) increase in the mean score was observed in the post-test when compared to the pre-test. The mean increase in test scores was 46.25% (range, 26.10-83.83%) (Table 1).

The number of students who completed the end-of-course survey was 439 (30.1%). The characteristics of the participants are summarized in Table 2. Only 90 (20.50%) participants believed that there are enough online radiology courses for medical students. Of all participants, 358 (81.55%) and 340 (77.45%) found online training either *extremely convenient* or *very convenient* and *extremely effective* or *very effective*, respectively. The Zoom technology platform used during the program was found to be *very friendly* or *extremely friendly* by 410 (93.41%) students.

**Table 2:** Survey participants’ characteristics

Characteristics	No. of Students
**Country**	
Turkey	436 (99.32%)
Other	3 (0.68%)
**Gender**	
Female	278 (63.33%)
Male	156 (35.54%)
Prefer not to answer	5 (1.14%)
**Year of medical school**	
First	83 (18.91%)
Second	85 (19.36%)
Third	86 (19.59%)
Fourth	83 (18.91%)
Fifth	50 (11.39%)
Sixth	30 (6.83%)
Other	22 (5.01%)
**Language of Education**	
Turkish	258 (58.77%)
English	176 (40.09%)
Other	5 (1.14%)
**Radiology Clerkship**^a^	
Exists	358 (81.55%)
Does not exist	81 (18.45%)

^a^Number of students attending a medical school that has a radiology clerkship exists or does not exist

Of the 439 participants, 415 (94.53%) attended all the sessions. The number of sessions attended by the remaining 24 (5.47%) students varied between 3 and 8. Participants were asked to rate each of the nine online sessions on a scale of 1 to 4: poor {1}, fair {2}, good {3}, and excellent {4}. The weighted average of these scores varied from 3.19 to 3.65, with an average score of 3.46 points. More than 92% of the participants indicated that they *strongly agree* or *agree* that the instructor’s communication was clear and easy to understand.

**Fig. 1:**
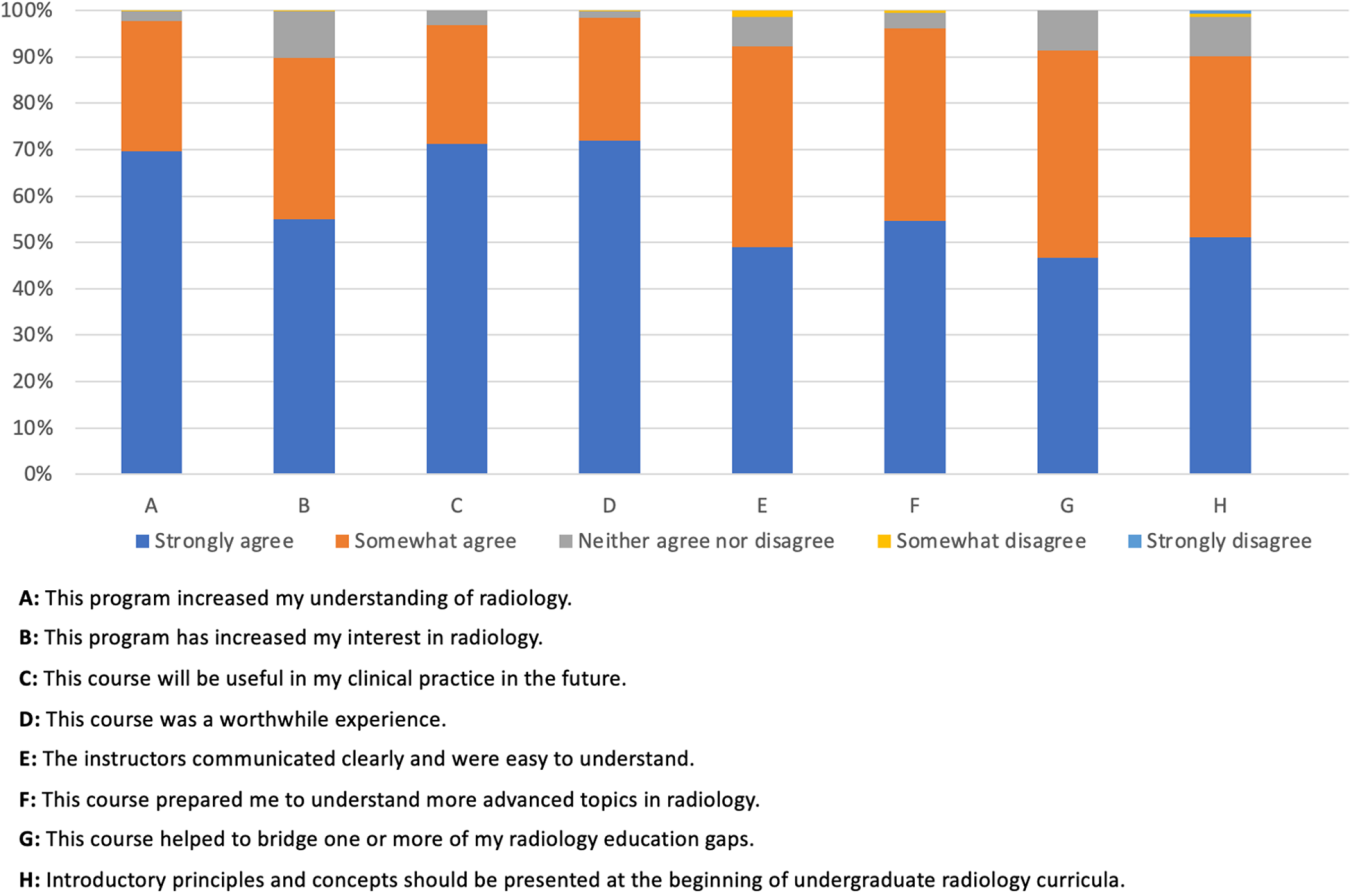
Course survey results

Of the 439 participants, 313 (71.30%), 306 (69.70%), and 316 (71.98%) indicated that they *strongly agree* that the course would be useful in their clinical practice in the future, the course increased their understanding of radiology, and the course was a worthwhile experience. Also, a total of 224 (51.03%) and 172 (39.18%) participants indicated that they *strongly agree* and *somewhat agree*, respectively, that introductory principles and concepts should be presented at the beginning of the undergraduate radiology curriculum. Figure 1 summarizes responses to the eight survey questions.

Participants were also asked to rate their level of confidence before and after the course on a scale of 1–4: not confident at all {1}, somewhat confident {2}, moderately confident {3}, and very confident {4}. On a weighted average, the confidence level before the course was 1.83 (range,1.68–1.93), while the level of confidence after the course was 3.09 (range, 2.95–3.29). The change in the calculated average level of confidence demonstrates a 68% increase after the course (Table 3). Almost all participants (99.32%) agreed that the program either met or exceeded their expectations. Two hundred and forty-one (54.90%) of the students indicated that the course increased their interest in radiology, and 248 (56.49%) were considering radiology as a specialty.

**Table 3:** Level of confidence before and after the course

		1: Not confident at all	2: Somewhat confident	3: Moderately confident	4: Very confident	Weighted average
**I am familiar with the principles and concepts behind imaging modalities**	Before	36.45%	38.50%	20.50%	4.56%	1.93
	After	0.23%	10.93%	48.52%	40.32%	3.29
**I am familiar with the indications of different imaging modalities**	Before	38.58%	34.93%	21.00%	5.48%	1.93
	After	0.23%	16.86%	50.34%	32.57%	3.15
**I am familiar with the history and evolution of radiology as a clinical field**	Before	49.09%	37.44%	10.05%	3.42%	1.68
	After	1.14%	22.55%	51.48%	24.83%	3.00
**I know how to approach and interpret an imaging study**	Before	47.49%	31.05%	16.21%	5.25%	1.79
	After	2.05%	24.89%	49.09%	23.97%	2.95

There was no statistically significant correlation between the attended sessions and year of studies in medicine or overall satisfaction with the course (*p* > .005).

Of 439 participants, 136 (31%) provided suggestions on ways to improve the program’s learning experience in the free text option.

## Discussion

Despite the fact that imaging is increasingly employed in everyday clinical practice, medical education in radiology continues to be undervalued in curricula across institutions worldwide [14]. Traditionally, radiology rotations have been based on passive learning through didactic lectures and empirical experience attained by accompanying radiologists in the reading room [15]. Increased workload, interruptions, complicated clinical situations, and the rotation schedules of attending radiologists, which often restrict the possibility of supervised learning, are all common obstacles to this sort of learning [15–17]. General learners frequently prefer a radiology curriculum that is well-integrated and includes small-group sessions and case-based learning [18, 19]. The ongoing COVID-19 pandemic, as well as the educational demands of the medical student audience, necessitate a shift in traditional teaching methods. Luckily, modern learners have access to a broad range of internet resources that enable them to work from home and learn from esteemed physicians throughout the nation while adhering to social distancing standards. The digital world is a continuously developing platform that can engage students in a variety of ways, including but not limited to 3D virtual environments [20], web-based educational tools [21], and social networking platforms that allow for mass communication [22].

Our radiology concepts course was designed to meet the needs of medical students and expand their access to online education in order to take a step towards universalizing radiology knowledge. Webinars are becoming more popular because of their convenience and cost-effectiveness, and they seem to be on their way to becoming one of the most essential instructional tools of the future [23]. These online learning technologies, in contrast to traditional didactic environments, provide for greater ease of access, expanded networking options for students and radiologists, and broader transmission of education. We can raise interest in radiology as a discipline and as a prospective profession for medical students across the world by providing access to instructional tools and networking opportunities with educators worldwide.

In our Principles of Radiology course, to promote audience participation, we used Zoom’s screen share, live polling, and chat box features. Students were able to contribute to the discussions during our lectures and ask questions via live chat. At the conclusion of the webinars, our moderators monitored and transmitted the questions to the speaker(s). This allowed for more engaging lectures and created a highly welcoming environment for our audience. Furthermore, the webinar lectures were recorded and published so that our students had the option to watch them at their convenience.

Overall, we had a higher-than-expected level of participation in our online course, including 1,458 registrants, 506 attendants, and 439 survey respondents from 41 different universities across Turkey. Almost 80% of the respondents said they believed that there were not enough online radiology courses for medical students at their current institutions; 81.55% and 77.45% found online training either *extremely* or *very convenient* and *extremely* or *very effective*, respectively. The technology platforms used were found to be *very* or *extremely friendly* by 93.42% of the respondents. Finally, around 80% found the online course *very* or *extremely convenient* and *effective*. Above 90% percent of respondents said they *strongly agree* or *somewhat agree* that introductory principles and concepts in radiology should be taught earlier in medical education. These findings highlight the underrepresentation of radiology education in medical school curricula and the need for new virtual learning platforms in radiology for medical students.

The demographic data generated from our polled participants revealed that 56.49% were already considering radiology as a career option after medical school. However, the student sample was likely heavily biased toward individuals who were already committed to pursuing a career in radiology or rather satisfied with the offer.

The post-test results showed a substantial (46.25%; *p* < .001) improvement over the pre-test scores, indicating that the webinar content improved their grasp of the topics. The program received an overall positive response, with around 90% or more of responders saying that they either *somewhat agree* or *strongly agree* that it improved their comprehension of radiology, piqued their interest in radiology, and will be valuable in their clinical practice in the future.

In general, respondents *very strongly agree* that the training was valuable, and almost all respondents (99.32%) agreed that it met or surpassed their expectations. The curriculum boosted respondents’ confidence in their understanding of the ideas underpinning imaging modalities, indications for imaging modalities, radiology’s history, and evolution, and how to interpret an imaging study. These data indicate that this virtual program and others like it can have a strongly favorable influence on medical student education worldwide.

To encourage even more audience involvement, the remote-control option on Zoom, which can mirror the radiologist’s experience of evaluating cases with a consulting physician, might be utilized in the future. While this adjustment has been shown to benefit resident learners [24], it has the potential to benefit medical student learners as well. This could be a very efficient way of teaching small groups, although it might not be as convenient in large groups of learners. Medical students have previously shown satisfaction with learning using virtual workstations, which simulate the environment of working radiologists [14, 17]. Several students sought a more thorough comprehension of core topics prior to the webinar, such as a review of suggested books before each session. This structure would be similar to the flipped classroom concept, which allows students to examine content at their own leisure before applying it during the webinar lectures [16]. Thus, it increases audience engagement and improves the higher-order cognitive grasp of the topic by shifting the webinar delivery schedule to encourage active learning.

Even if the course is designed as an alternative addendum, each student was asked to share her opinions about the consistency and benefits of this course relative to the already ongoing curriculum.

There are some limitations of our study. First, only a small proportion of the medical students in Turkey were registered and approximately 1/3 of them took part in both proficiency tests. Second, only %30 of the students filled out the final questionnaire. Third, there was no control group. Finally, answers to the post-test questions were gathered immediately after the lessons. Thus, this could create a short-term memory false positivity even if we wanted to overcome it by assessing multiple different learning objectives in each session.

## Conclusion

While webinars have been around as an instructional tool for a long time, they are currently one of the few ways to get medical education in the face of the COVID-19 pandemic, and they have the potential to determine the future of medical education. In the long run, it is unknown how these educational approaches will alter medical student behavior and attitudes regarding online learning versus traditional in-person didactics. Regardless, over the course of 3 months, we could already see the benefits of the webinars in improving radiological learning methods. In order to truly understand the effectiveness of these methods in the scope of future radiology education, it is crucial to assess medical students’ feedback and improvements in radiology knowledge gained through virtual education in the post-COVID age.

## Supplementary Information

**Additional file 1.** 

## Data Availability

All data generated or analysed during this study are included in this published article and its supplementary information files.

## Abbreviations

ESR: European Society of Radiology
IRB: Institutional review board
