# Supplementary material for: Knowing the ABCs: teaching the principles of radiology to medical students in Turkey
Source: BMC Med Educ. 2022 Dec 12;22:857. doi: 10.1186/s12909-022-03885-8 (PMC9742646; doi:10.1186/s12909-022-03885-8)
Supplement: Supplementary file 1 — Additional file 1. [file 12909_2022_3885_MOESM1_ESM.pdf]

# Supplementary Figure 1
# Please indicate your gender

Answered: 439 Skipped: 0

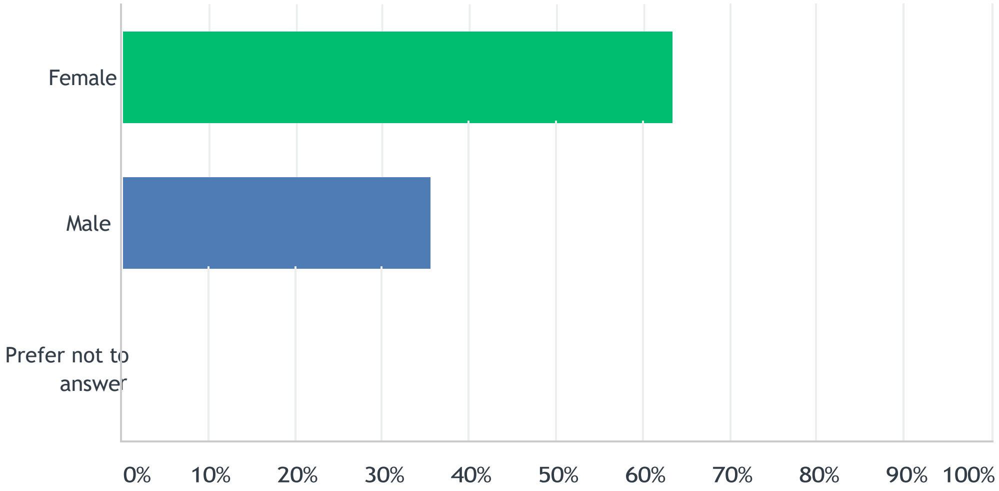


| ANSWER CHOICES | RESPONSES | |
|---|---|---|
| Female | 63.33% | 278 |
| Male | 35.54% | 156 |
| Prefer not to answer | 1.14% | 5 |
| TOTAL | | 439 |

# Supplementary Figure 2

## What is the language used to teach most of the medical curriculum at your current medical school?

Answered: 439 Skipped: 0

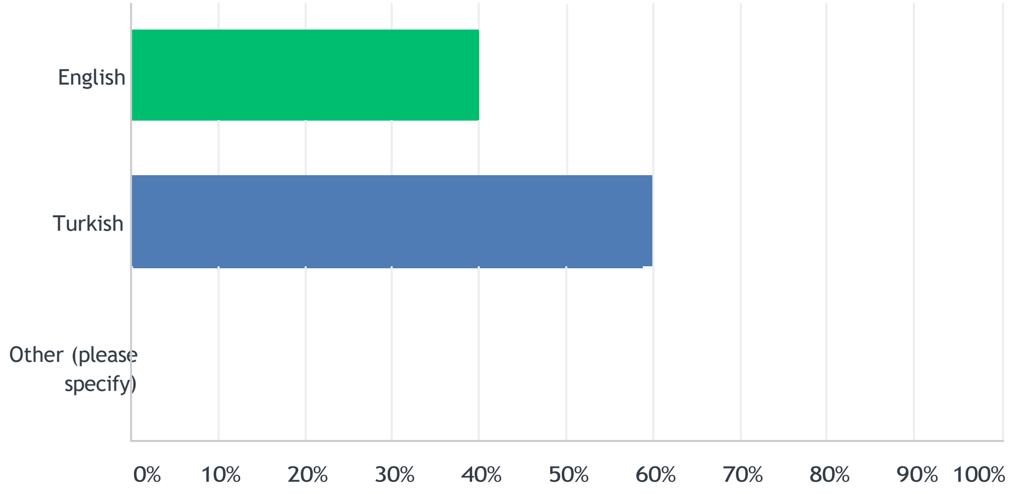


| ANSWER CHOICES | RESPONSES | |
|---|---|---|
| English | 40.09% | 176 |
| Turkish | 58.77% | 258 |
| Other (please specify) | 1.14% | 5 |
| TOTAL | | 439 |

# Supplementary Figure 3
## What class year are you as of the 2019-2020 school year?

Answered: 439 Skipped: 0

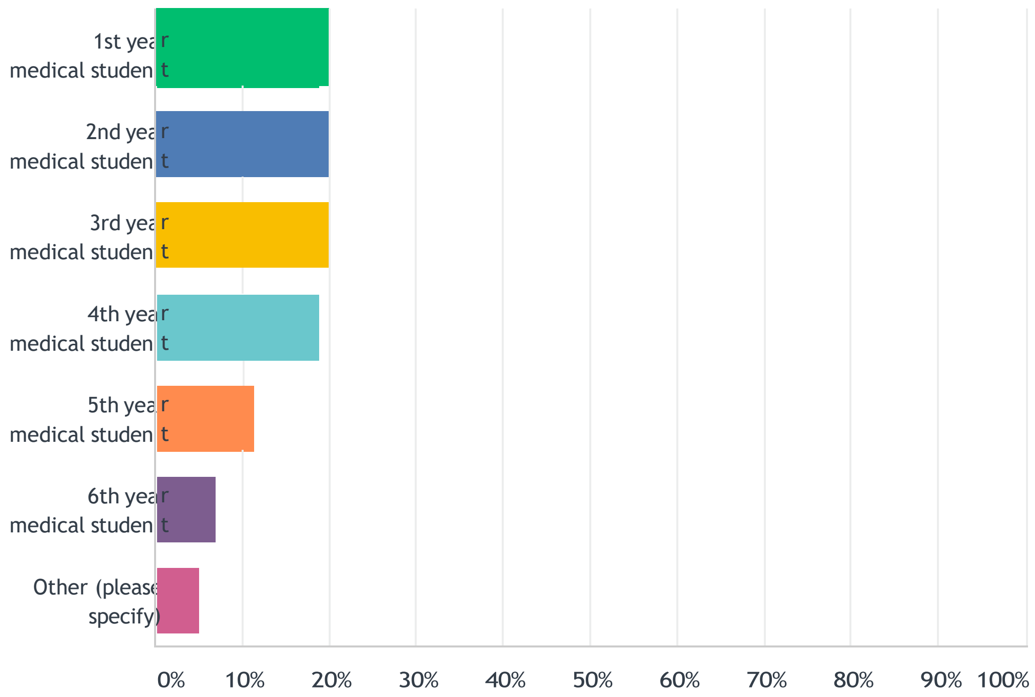


| ANSWER CHOICES | RESPONSES | |
|---|---|---|
| 1st year medical student | 18.91% | 83 |
| 2nd year medical student | 19.36% | 85 |
| 3rd year medical student | 19.59% | 86 |
| 4th year medical student | 18.91% | 83 |
| 5th year medical student | 11.39% | 50 |
| 6th year medical student | 6.83% | 30 |
| Other (please specify) | 5.01% | 22 |
| TOTAL | | 439 |

## Supplementary Figure 4
## In what country do you study?

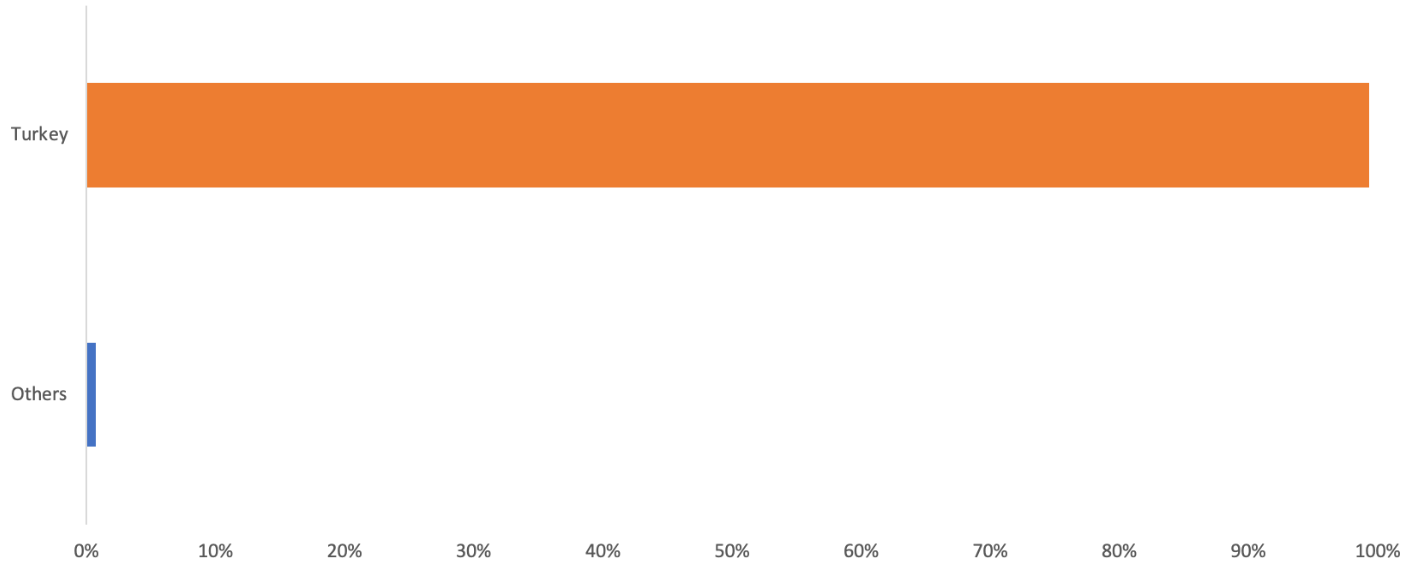


Answered: 439 Skipped: 0

| ANSWER CHOICES | RESPONSES | |
|---|---|---|
| Turkey | 99.32% | 436 |
| Others | 0.68% | 3 |
| TOTAL | | 439 |

# Supplementary Figure 5

## Please specify the following regarding where do you study

Answered: 416    Skipped: 23

| ANSWER CHOICES | RESPONSES | |
|---|---|---|
| City | 100.00% | 416 |
| Provenance | 66.35% | 276 |

# Supplementary Figure 6
# Is a radiology clerkship required at your medical school?

Answered: 439 Skipped: 0

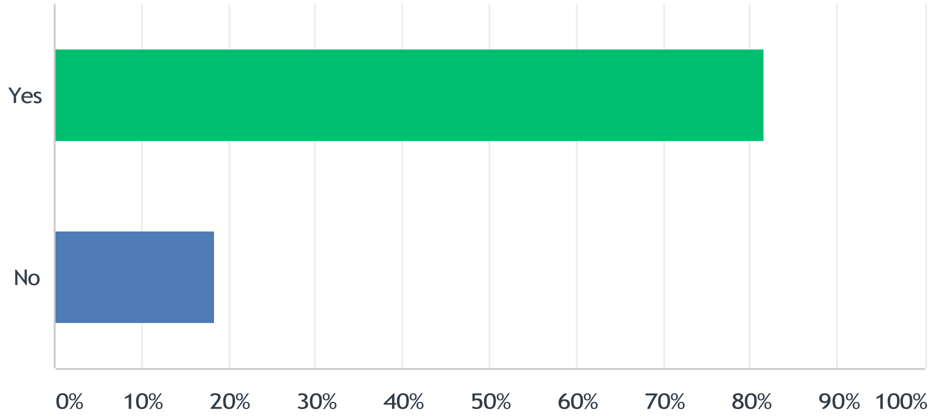


| ANSWER CHOICES | RESPONSES | |
|---|---|---|
| Yes | 81.55% | 358 |
| No | 18.45% | 81 |
| TOTAL | | 439 |

# Supplementary Figure 7
# What model of radiology education do your school offer?

Answered: 439    Skipped: 0

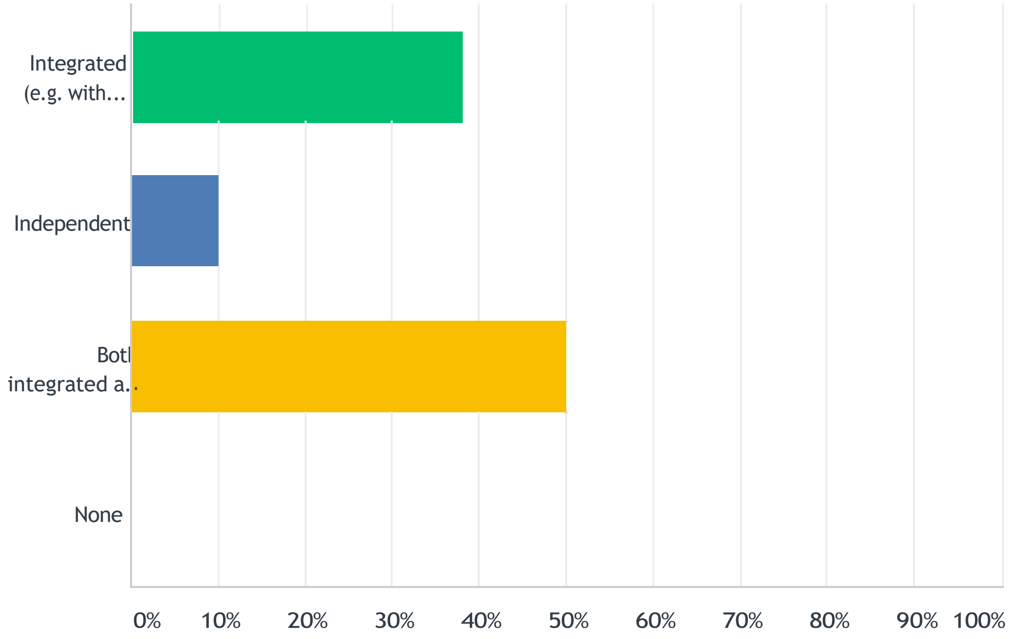


| ANSWER CHOICES | RESPONSES | |
|---|---|---|
| Integrated (e.g. with anatomy, pathology, etc) | 38.04% | 167 |
| Independent | 10.25% | 45 |
| Both integrated and independent | 50.11% | 220 |
| None | 1.59% | 7 |
| TOTAL | | 439 |

# Supplementary Figure 8

## How much time did you spend in a traditional in-person radiology clerkship?

Answered: 439    Skipped: 0

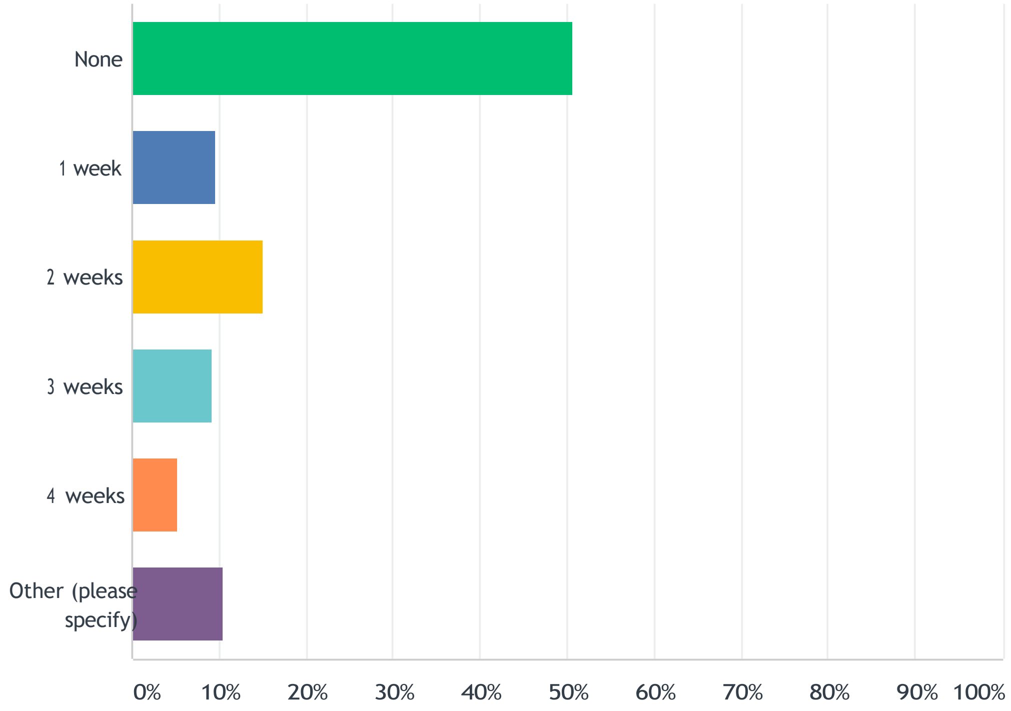


| ANSWER CHOICES | RESPONSES | |
|---|---|---|
| None | 50.57% | 222 |
| 1 week | 9.57% | 42 |
| 2 weeks | 15.03% | 66 |
| 3 weeks | 9.11% | 40 |
| 4 weeks | 5.24% | 23 |
| Other (please specify) | 10.48% | 46 |
| TOTAL | | 439 |

# Supplementary Figure 9
# How many sessions of this course have you watched?

Answered: 439    Skipped: 0

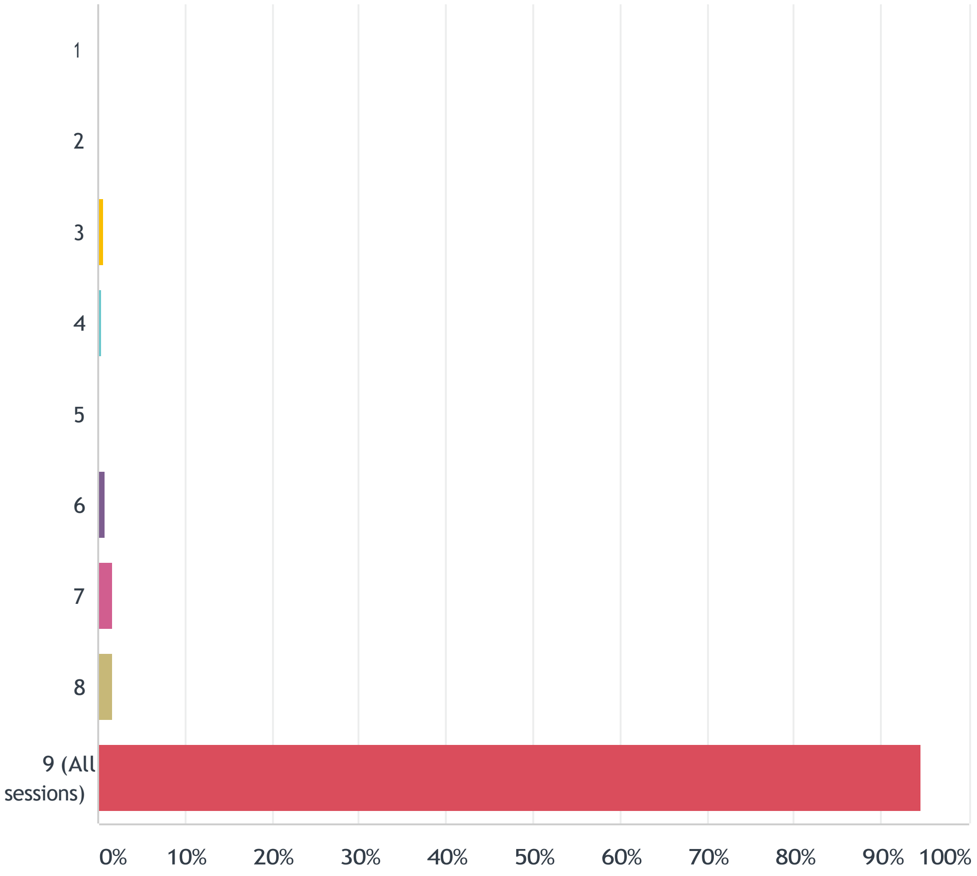


| ANSWER CHOICES | RESPONSES | |
|---|---|---|
| 1 | 0.00% | 0 |
| 2 | 0.00% | 0 |
| 3 | 0.68% | 3 |
| 4 | 0.46% | 2 |
| 5 | 0.23% | 1 |
| 6 | 0.91% | 4 |
| 7 | 1.59% | 7 |
| 8 | 1.59% | 7 |
| 9 (All sessions) | 94.53% | 415 |
| TOTAL | | 439 |

# Supplementary Figure 10

## If applicable, mention the reason(s) that made you skip some of the sessions

Answered: 47 Skipped: 392

# Supplementary Figure 11
## This program increased your understanding of radiology

Answered: 439    Skipped: 0

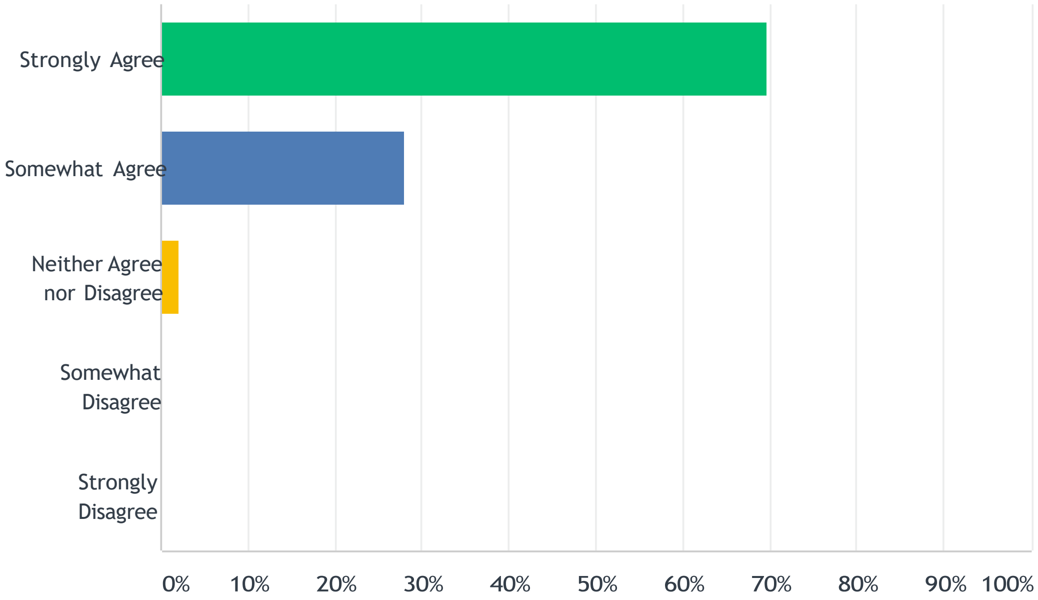


| ANSWER CHOICES | RESPONSES | |
|---|---|---|
| Strongly Agree | 69.70% | 306 |
| Somewhat Agree | 28.02% | 123 |
| Neither Agree nor Disagree | 2.05% | 9 |
| Somewhat Disagree | 0.23% | 1 |
| Strongly Disagree | 0.00% | 0 |
| TOTAL | | 439 |

# Supplementary Figure 12
# This program has increased your interest in radiology.

Answered: 439 Skipped: 0

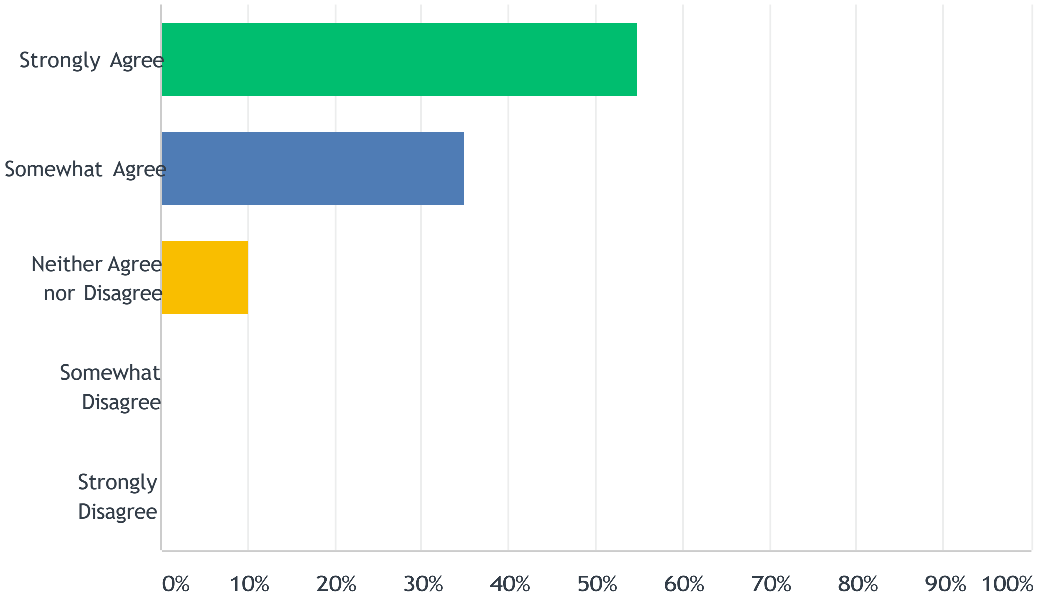


| ANSWER CHOICES | RESPONSES | |
|---|---|---|
| Strongly Agree | 54.90% | 241 |
| Somewhat Agree | 34.85% | 153 |
| Neither Agree nor Disagree | 10.02% | 44 |
| Somewhat Disagree | 0.23% | 1 |
| Strongly Disagree | 0.00% | 0 |
| TOTAL | | 439 |

# Supplementary Figure 13
# Are you considering radiology as a specialty?

Answered: 439 Skipped: 0

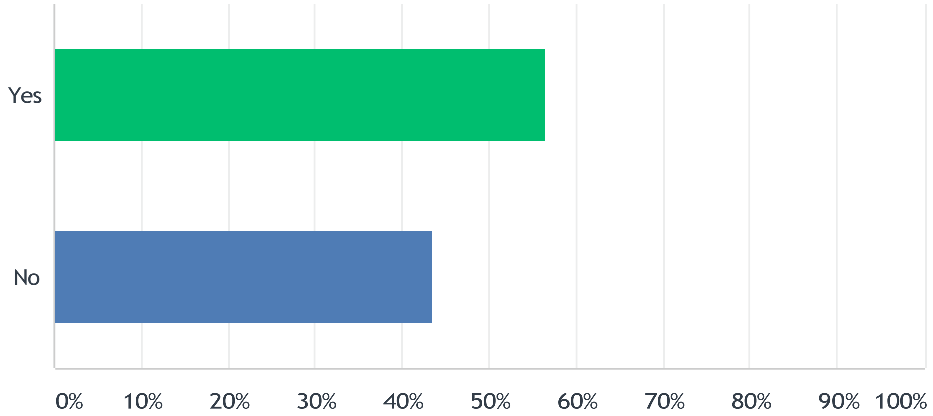


| ANSWER CHOICES | RESPONSES | |
|---|---|---|
| Yes | 56.49% | 248 |
| No | 43.51% | 191 |
| TOTAL | | 439 |

# Supplementary Figure 14
## This course will be useful in my clinical practice in the future

Answered: 439 Skipped: 0

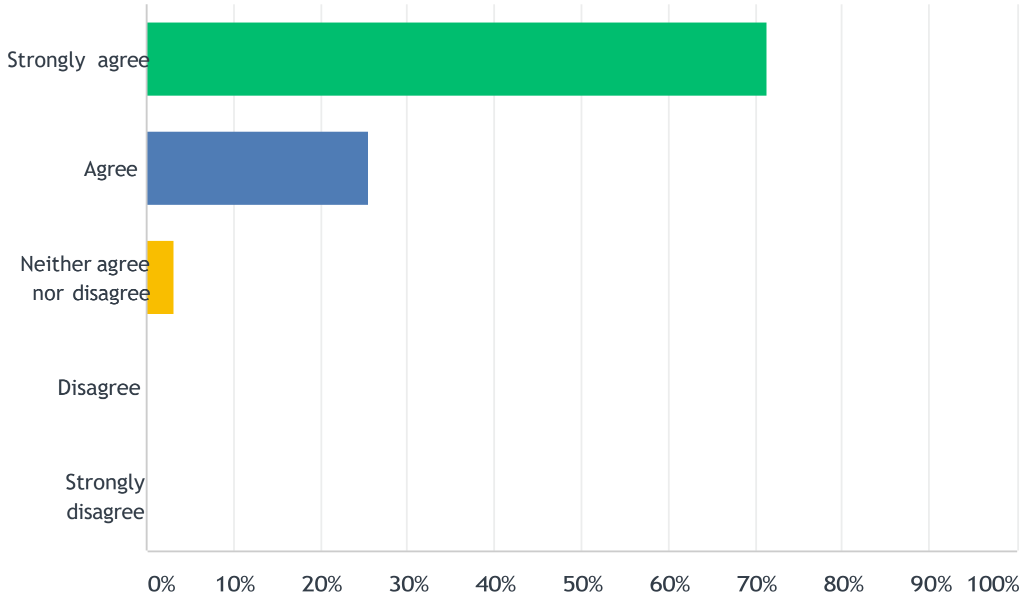


| ANSWER CHOICES | RESPONSES | |
|---|---|---|
| Strongly agree | 71.30% | 313 |
| Agree | 25.51% | 112 |
| Neither agree nor disagree | 3.19% | 14 |
| Disagree | 0.00% | 0 |
| Strongly disagree | 0.00% | 0 |
| TOTAL | | 439 |

# Supplementary Figure 15

## The amount of effort to complete the requirements for this program was:

Answered: 439 Skipped: 0

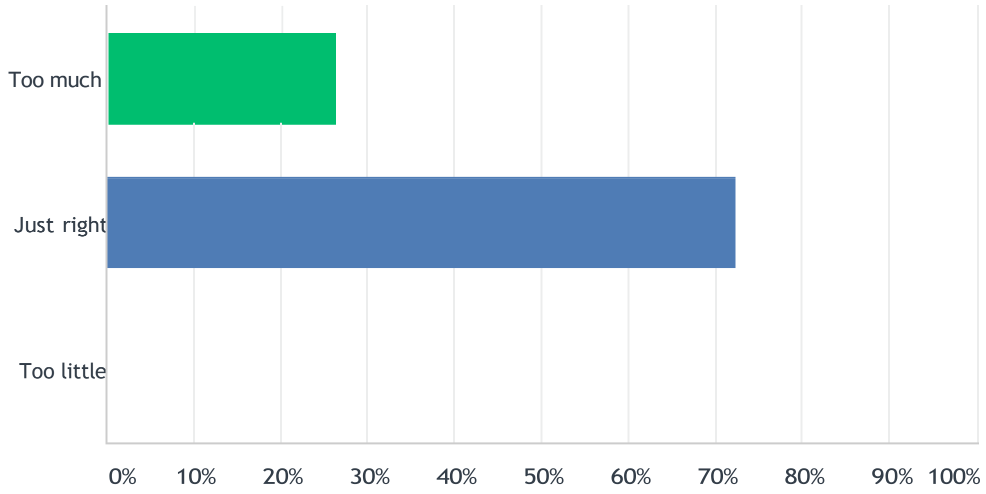


| ANSWER CHOICES | RESPONSES | |
|---|---|---|
| Too much | 26.42% | 116 |
| Just right | 72.44% | 318 |
| Too little | 1.14% | 5 |
| TOTAL | | 439 |

# Supplementary Figure 16

## Based on this course, what were the most important components in enhancing your understanding of radiology (Select all that apply)

Answered: 439 Skipped: 0

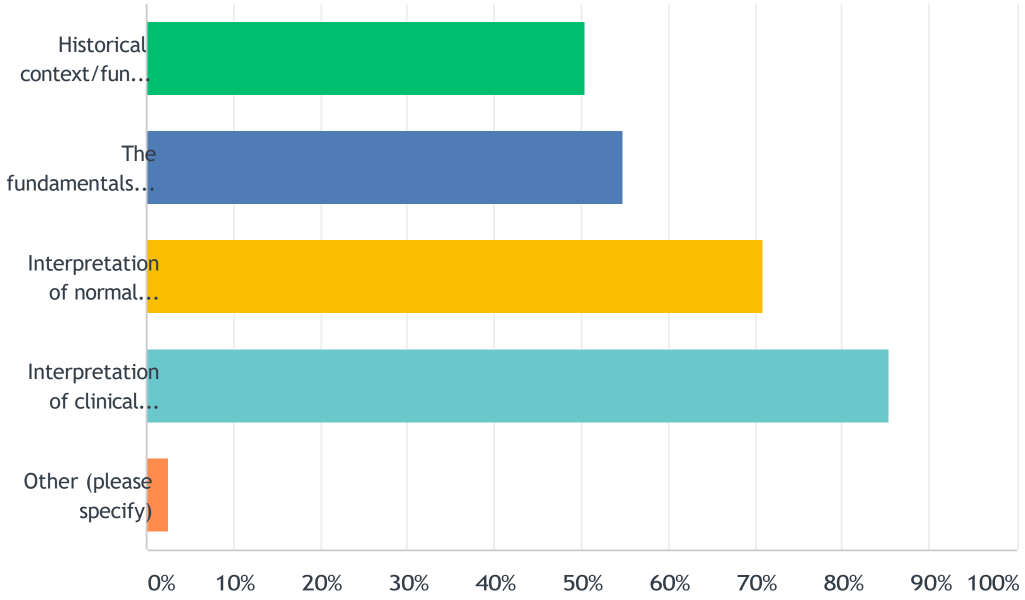


| ANSWER CHOICES | RESPONSES | |
|---|---|---|
| Historical context/fun facts | 50.34% | 221 |
| The fundamentals of physics and basic science | 54.90% | 241 |
| Interpretation of normal imaging | 70.84% | 311 |
| Interpretation of clinical cases | 85.42% | 375 |
| Other (please specify) | 2.51% | 11 |
| Total Respondents: 439 | | |

# Supplementary Figure 17
# This course was a worthwhile experience:

Answered: 439    Skipped: 0

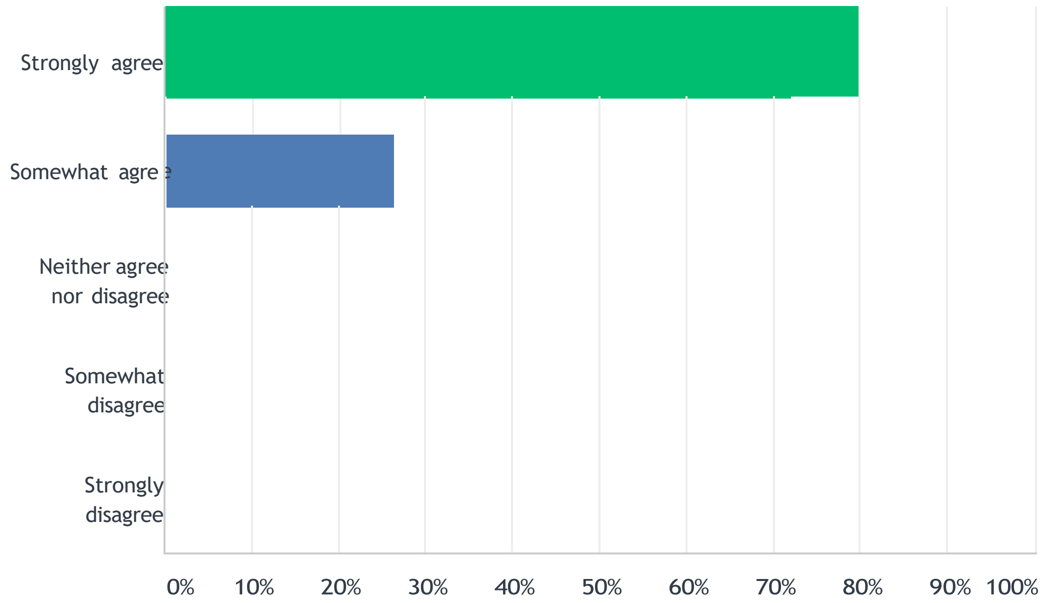


| ANSWER CHOICES | RESPONSES | |
|---|---|---|
| Strongly agree | 71.98% | 316 |
| Somewhat agree | 26.42% | 116 |
| Neither agree nor disagree | 1.37% | 6 |
| Somewhat disagree | 0.23% | 1 |
| Strongly disagree | 0.00% | 0 |
| TOTAL | | 439 |

# Supplementary Figure 18

## The instructors communicated clearly and were easy to understand.

Answered: 439 Skipped: 0

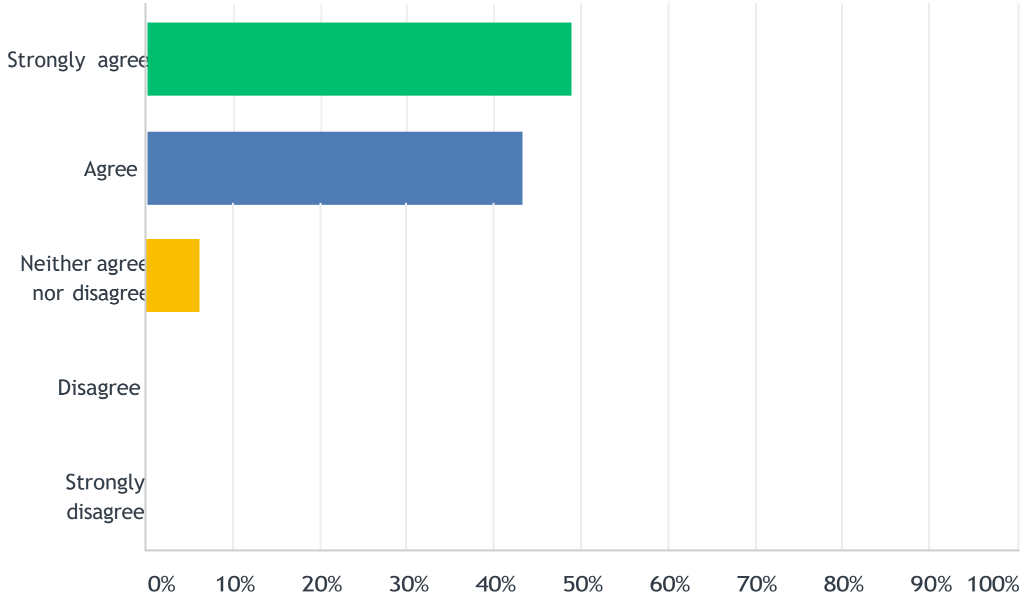


| ANSWER CHOICES | RESPONSES | |
|---|---|---|
| Strongly agree | 48.97% | 215 |
| Agree | 43.28% | 190 |
| Neither agree nor disagree | 6.38% | 28 |
| Disagree | 1.37% | 6 |
| Strongly disagree | 0.00% | 0 |
| TOTAL | | 439 |

# Supplementary Figure 19
# Please evaluate the sessions you attended.

Answered: 439 Skipped: 0

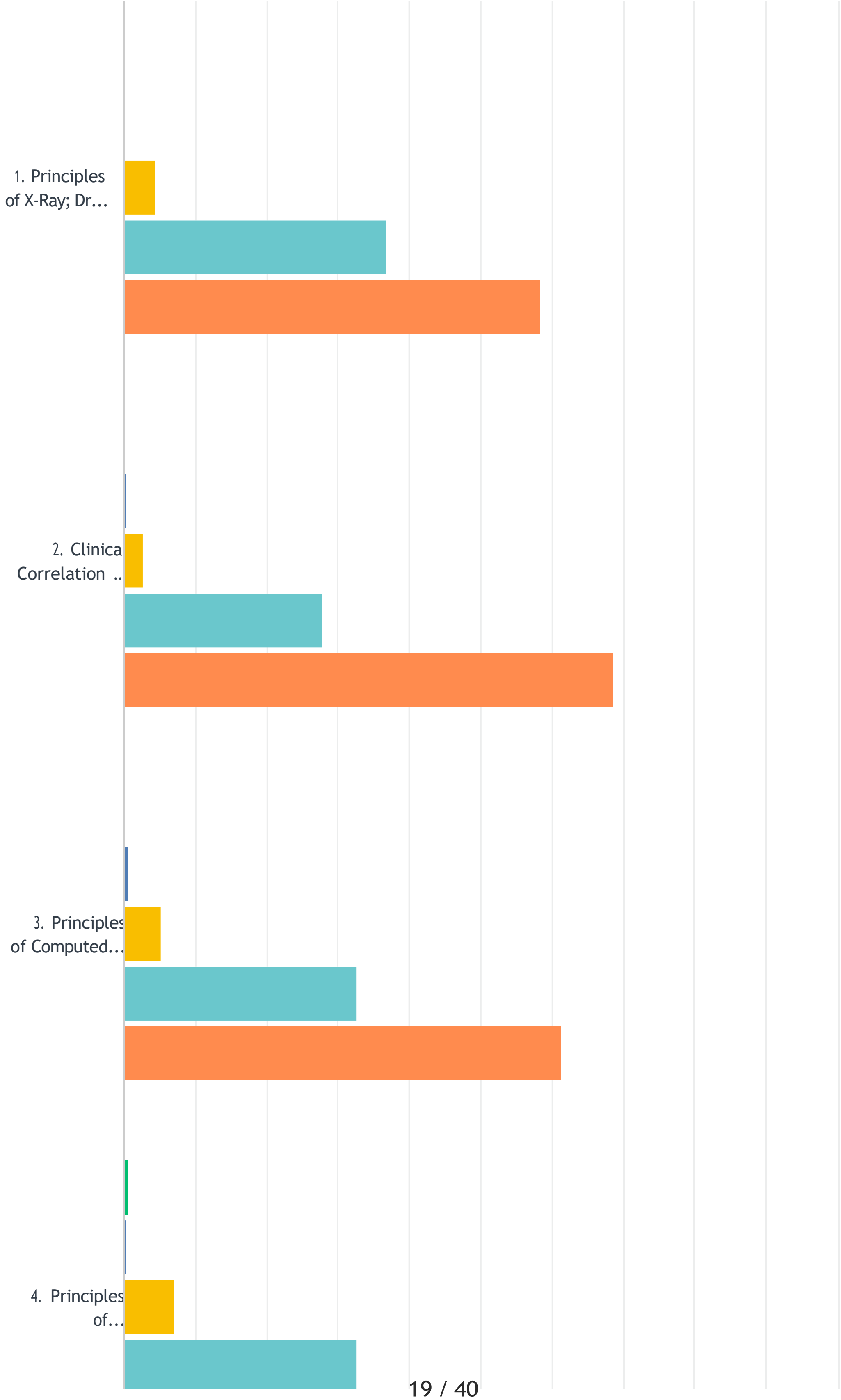

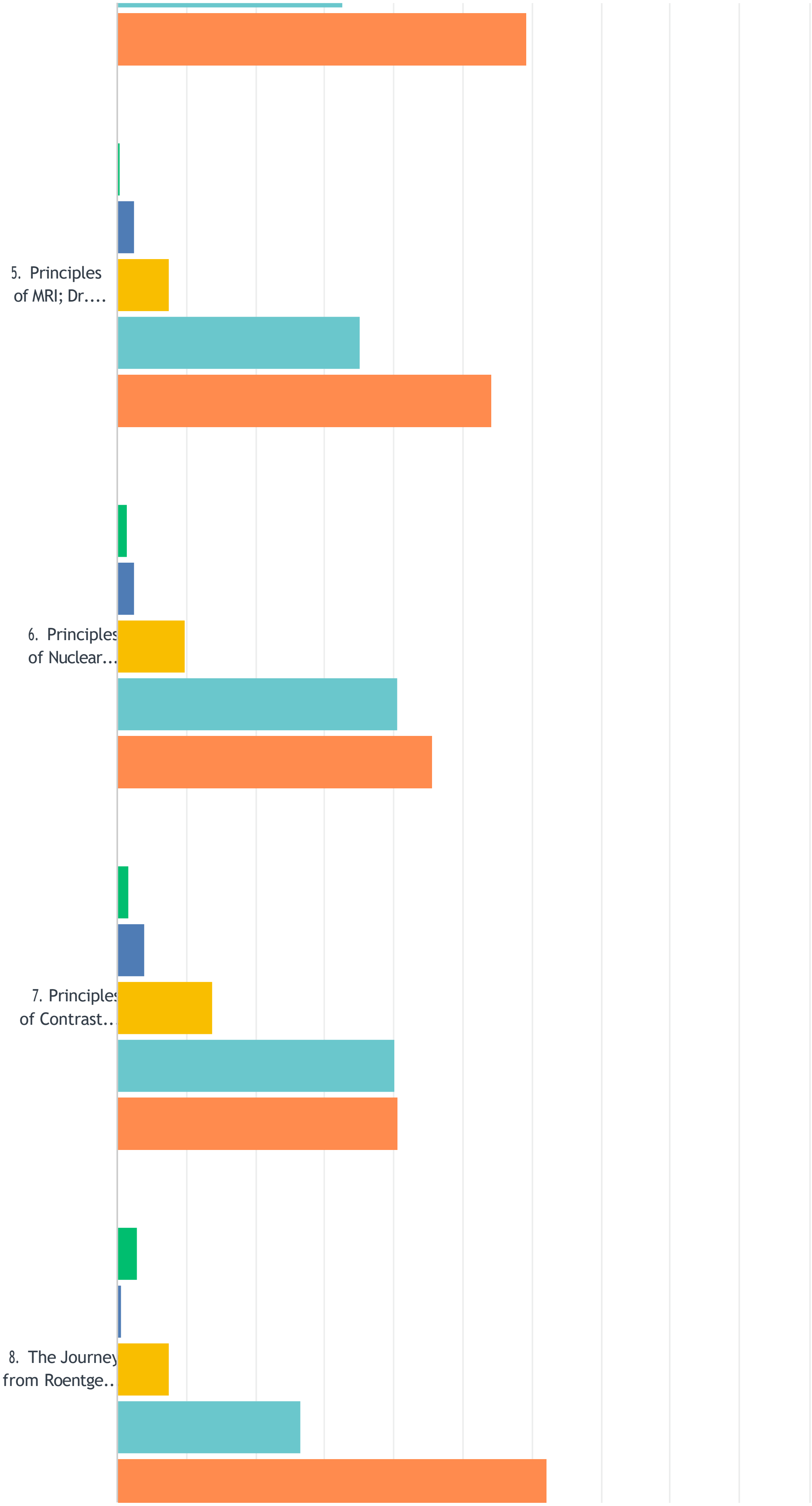
5. Principles of MRI; Dr....
6. Principles of Nuclear...
7. Principles of Contrast...
8. The Journey from Roentge...

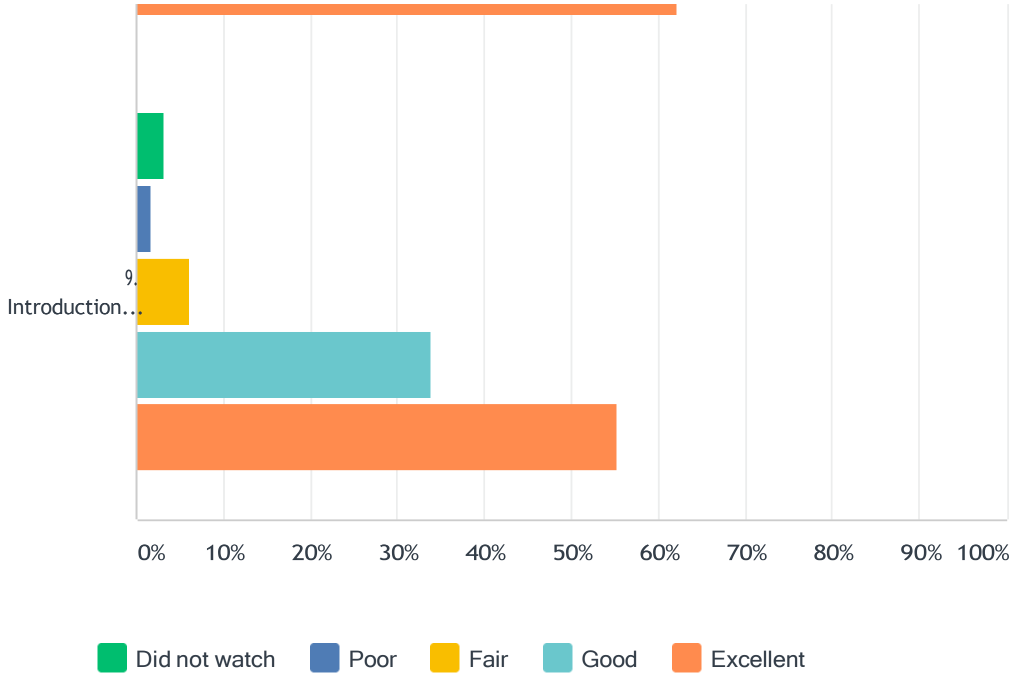


| | | | | | | | |
|---|---|---|---|---|---|---|---|
| 1. Principles of X-Ray; Dr. Ayman Gaballah | 0.23%<br>1 | 0.23%<br>1 | 4.34%<br>19 | 36.76%<br>161 | 58.45%<br>256 | 438 | 4.53 |
| 2. Clinical Correlation of the Chest Radiographs | 0.23%<br>1 | 0.46%<br>2 | 2.74%<br>12 | 27.85%<br>122 | 68.72%<br>301 | 438 | 4.64 |
| 3. Principles of Computed Tomography (CT); Dr. Khaled Elsayes | 0.23%<br>1 | 0.68%<br>3 | 5.24%<br>23 | 32.57%<br>143 | 61.28%<br>269 | 439 | 4.54 |
| 4. Principles of Ultrasonography; Dr. Aya Kamaya | 0.68%<br>3 | 0.46%<br>2 | 7.08%<br>31 | 32.65%<br>143 | 59.13%<br>259 | 438 | 4.49 |
| 5. Principles of MRI; Dr. Victoria Chernyak | 0.46%<br>2 | 2.53%<br>11 | 7.59%<br>33 | 35.17%<br>153 | 54.25%<br>236 | 435 | 4.40 |
| 6. Principles of Nuclear Medicine; Dr. Haitham Elsamaloty | 1.37%<br>6 | 2.51%<br>11 | 9.82%<br>43 | 40.64%<br>178 | 45.66%<br>200 | 438 | 4.27 |
| 7. Principles of Contrast Agents; Dr. Kedar Jambhekar | 1.59%<br>7 | 3.87%<br>17 | 13.90%<br>61 | 40.09%<br>176 | 40.55%<br>178 | 439 | 4.14 |
| 8. The Journey from Roentgen to Artificial Intelligence; Dr. Larry Stein | 2.96%<br>13 | 0.68%<br>3 | 7.52%<br>33 | 26.65%<br>117 | 62.19%<br>273 | 439 | 4.44 |

| | | | | | | | |
|---|---|---|---|---|---|---|---|
| 9. Introduction to Interventional Radiology | 3.20% 14 | 1.60% 7 | 6.16% 27 | 33.79% 148 | 55.25% 242 | [illegible] | [illegible] |

# Supplementary Figure 20
## What is the common factor(s) between the lectures you liked the most

Answered: 439 Skipped: 0

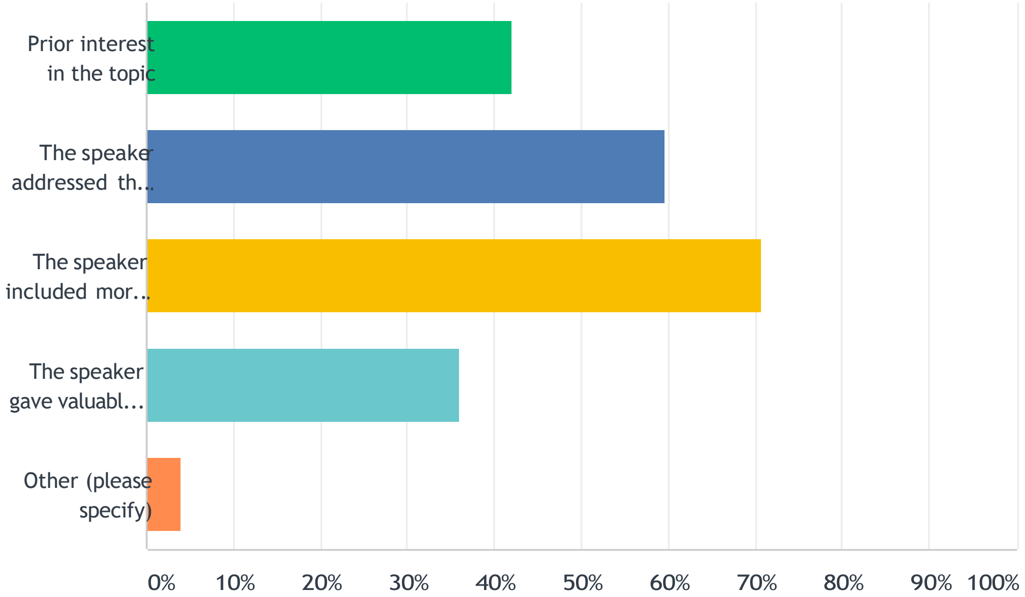


| ANSWER CHOICES | RESPONSES | |
|---|---|---|
| Prior interest in the topic | 42.14% | 185 |
| The speaker addressed the topic thoroughly | 59.68% | 262 |
| The speaker included more media (e.g. images, videos, animations) | 70.62% | 310 |
| The speaker gave valuable career advice | 35.99% | 158 |
| Other (please specify) | 3.87% | 17 |
| Total Respondents: 439 | | |

# Supplementary Figure 21
# How useful were the pretest and post-test?

Answered: 439 Skipped: 0

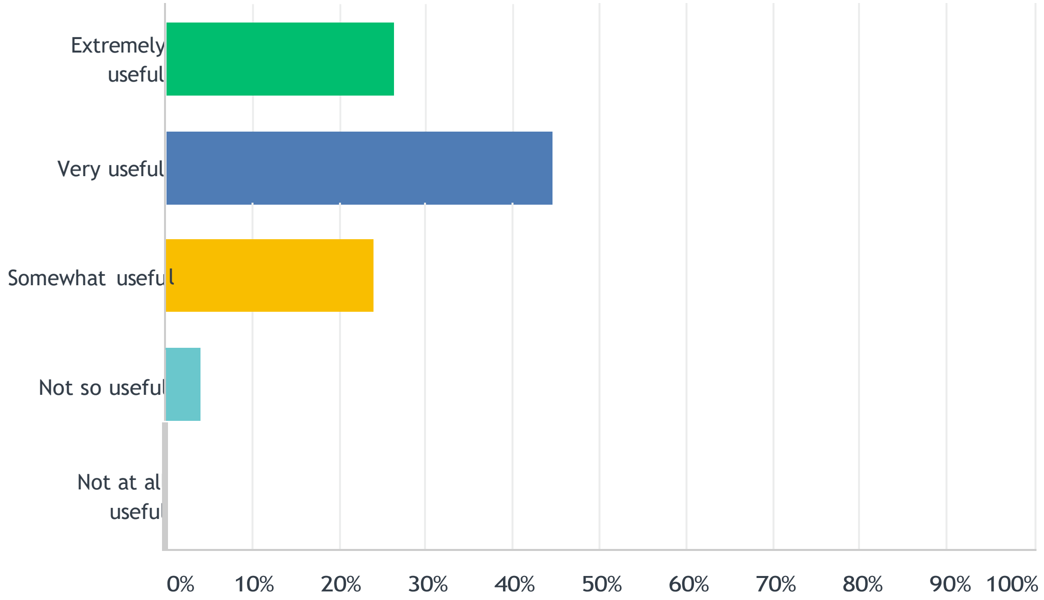


| ANSWER CHOICES | RESPONSES | |
|---|---|---|
| Extremely useful | 26.42% | 116 |
| Very useful | 44.65% | 196 |
| Somewhat useful | 24.15% | 106 |
| Not so useful | 4.10% | 18 |
| Not at all useful | 0.68% | 3 |
| TOTAL | | 439 |

# Supplementary Figure 22

## As a student, I found the topics presented in the educational sessions to be:

Answered: 439 Skipped: 0

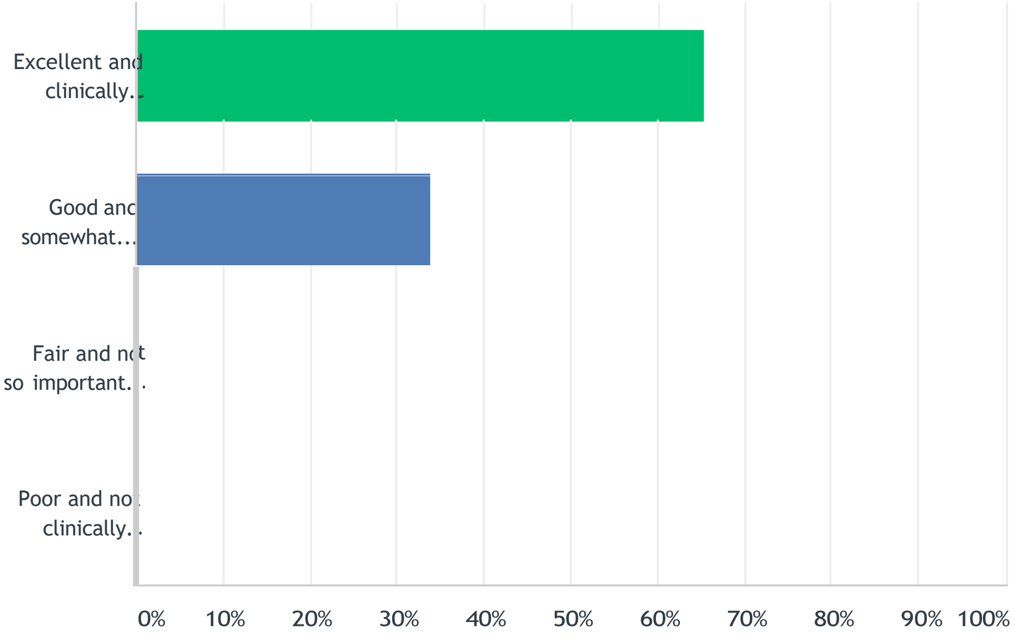


| ANSWER CHOICES | RESPONSES | |
|---|---|---|
| Excellent and clinically important | 65.38% | 287 |
| Good and somewhat important to learn | 33.94% | 149 |
| Fair and not so important to learn | 0.68% | 3 |
| Poor and not clinically important at all | 0.00% | 0 |
| TOTAL | | 439 |

# Supplementary Figure 23
## Did the program meet your expectations?

Answered: 439 Skipped: 0

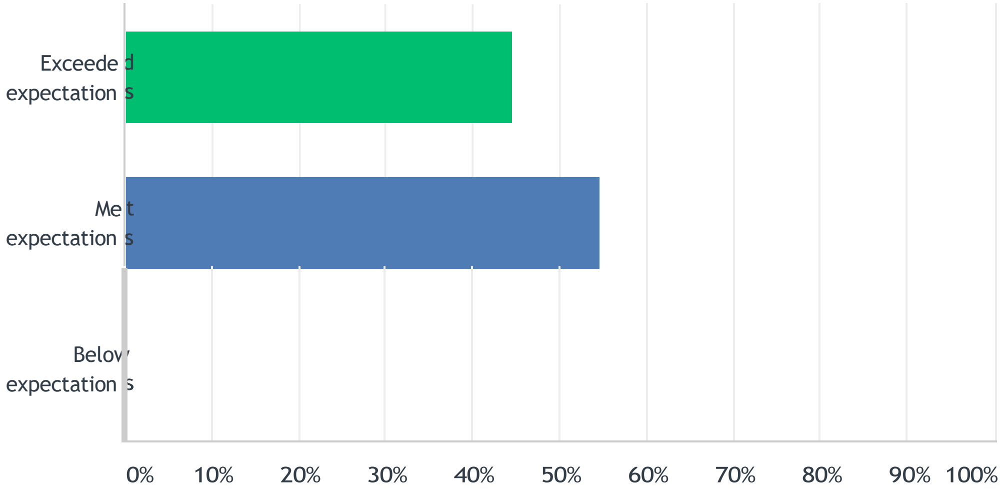


| ANSWER CHOICES | RESPONSES | |
|---|---|---|
| Exceeded expectations | 44.65% | 196 |
| Met expectations | 54.67% | 240 |
| Below expectations | 0.68% | 3 |
| TOTAL | | 439 |

## Supplementary Figure 24

## Please rate your confidence in the following BEFORE your participation in the program.

Answered: 439 Skipped: 0

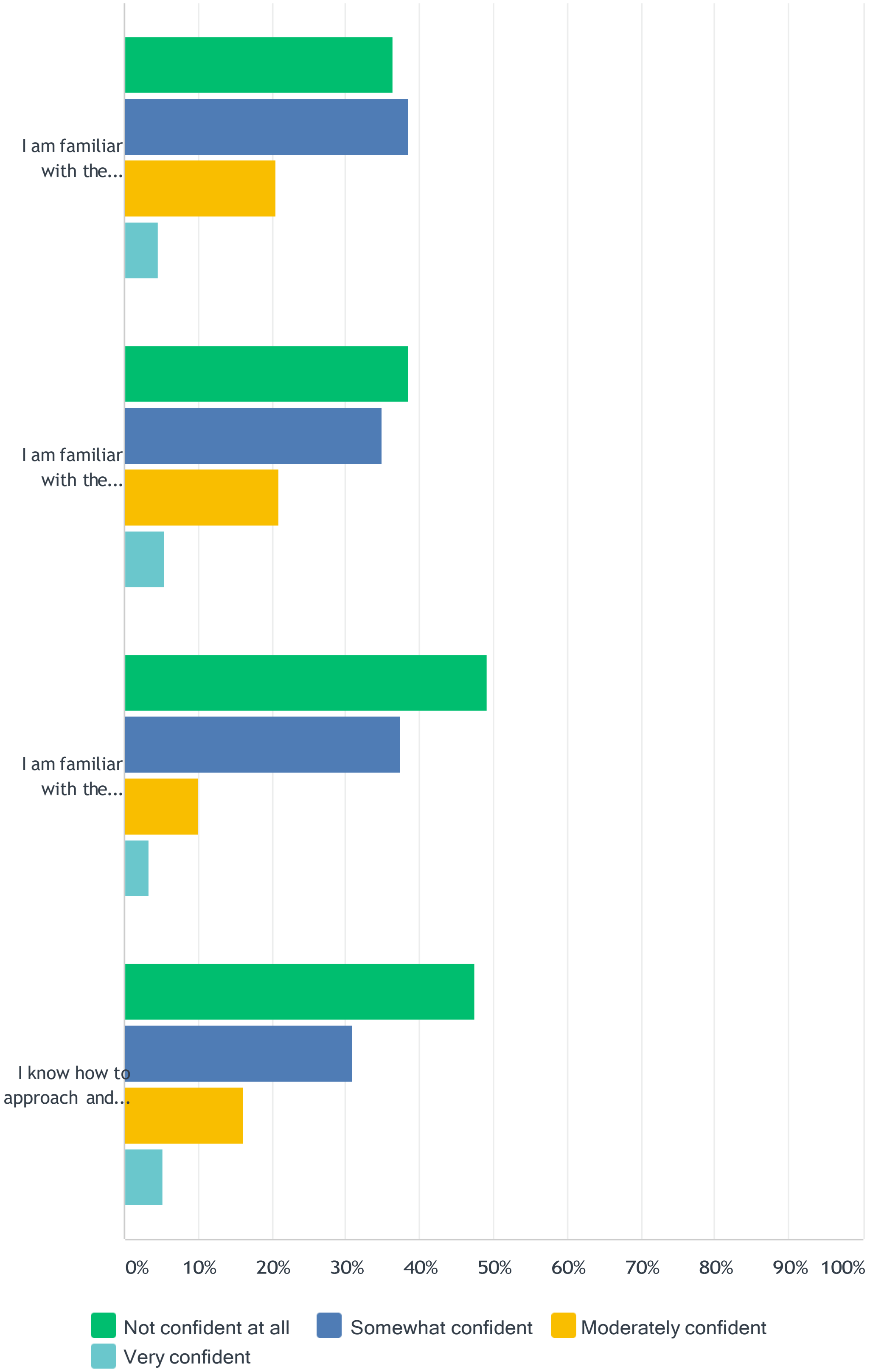

| | NOT CONFIDENT AT ALL | SOMEWHAT CONFIDENT | MODERATELY CONFIDENT | VERY CONFIDENT | TOTAL | WEIGHTED AVERAGE |
|---|---|---|---|---|---|---|
| I am familiar with the principles and concepts behind imaging modalities | 36.45%<br>160 | 38.50%<br>169 | 20.50%<br>90 | 4.56%<br>20 | 439 | 1.93 |
| I am familiar with the indications of different imaging modalities | 38.58%<br>169 | 34.93%<br>153 | 21.00%<br>92 | 5.48%<br>24 | 438 | 1.93 |
| I am familiar with the history and evolution of radiology as a clinical field | 49.09%<br>215 | 37.44%<br>164 | 10.05%<br>44 | 3.42%<br>15 | 438 | 1.68 |
| I know how to approach and interpret an imaging study | 47.49%<br>208 | 31.05%<br>136 | 16.21%<br>71 | 5.25%<br>23 | 438 | 1.79 |

# Supplementary Figure 25

## Please rate your confidence in the following AFTER your participation in the program.

Answered: 439 Skipped: 0

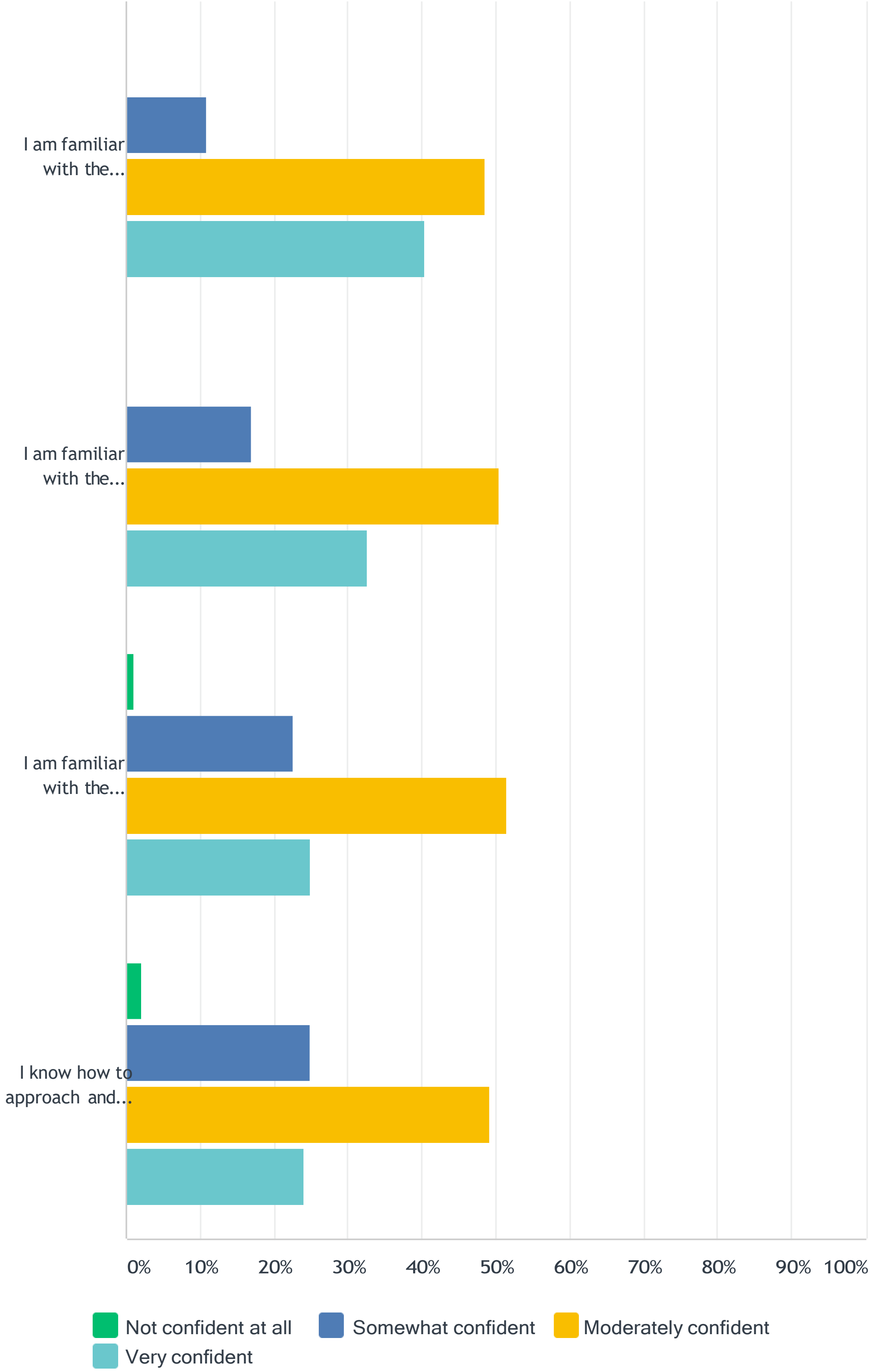

| | NOT CONFIDENT AT ALL | SOMEWHAT CONFIDENT | MODERATELY CONFIDENT | VERY CONFIDENT | TOTAL | WEIGHTED AVERAGE |
|---|---|---|---|---|---|---|
| I am familiar with the principles and concepts behind imaging modalities | 0.23%<br>1 | 10.93%<br>48 | 48.52%<br>213 | 40.32%<br>177 | 439 | 3.29 |
| I am familiar with the indications of different imaging modalities | 0.23%<br>1 | 16.86%<br>74 | 50.34%<br>221 | 32.57%<br>143 | 439 | 3.15 |
| I am familiar with the history and evolution of radiology as a clinical field | 1.14%<br>5 | 22.55%<br>99 | 51.48%<br>226 | 24.83%<br>109 | 439 | 3.00 |
| I know how to approach and interpret an imaging study | 2.05%<br>9 | 24.89%<br>109 | 49.09%<br>215 | 23.97%<br>105 | 438 | 2.95 |

# Supplementary Figure 26

## How do you rate your experience with the technological platforms we used to host the webinars, protests, and post-tests?

Answered: 439 Skipped: 0

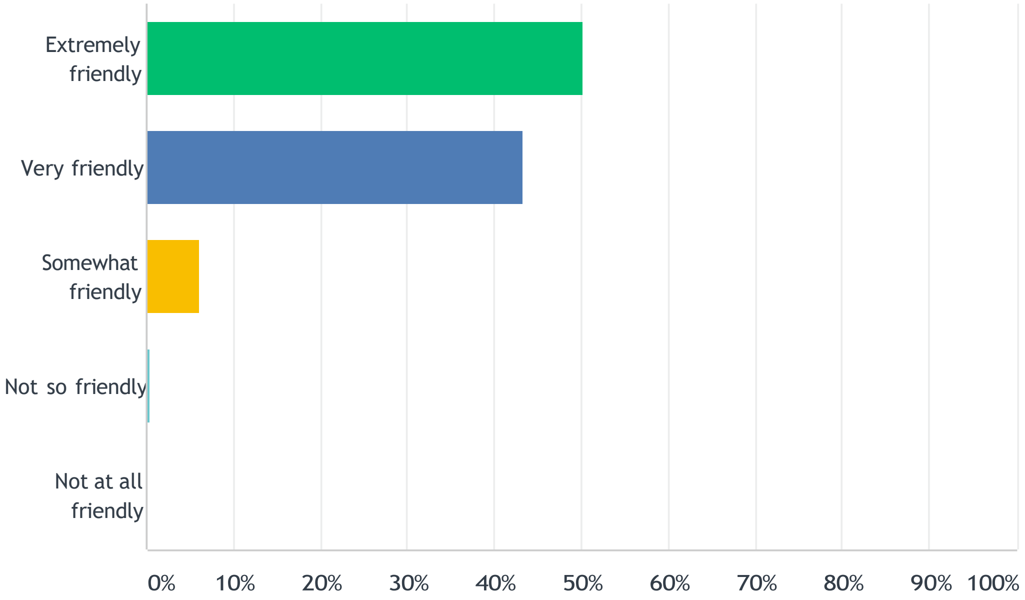


| ANSWER CHOICES | RESPONSES | |
|---|---|---|
| Extremely friendly | 50.11% | 220 |
| Very friendly | 43.28% | 190 |
| Somewhat friendly | 6.15% | 27 |
| Not so friendly | 0.46% | 2 |
| Not at all friendly | 0.00% | 0 |
| TOTAL | | 439 |

# Supplementary Figure 27

## Do you believe there are enough online radiology courses for medical students?

Answered: 439 Skipped: 0

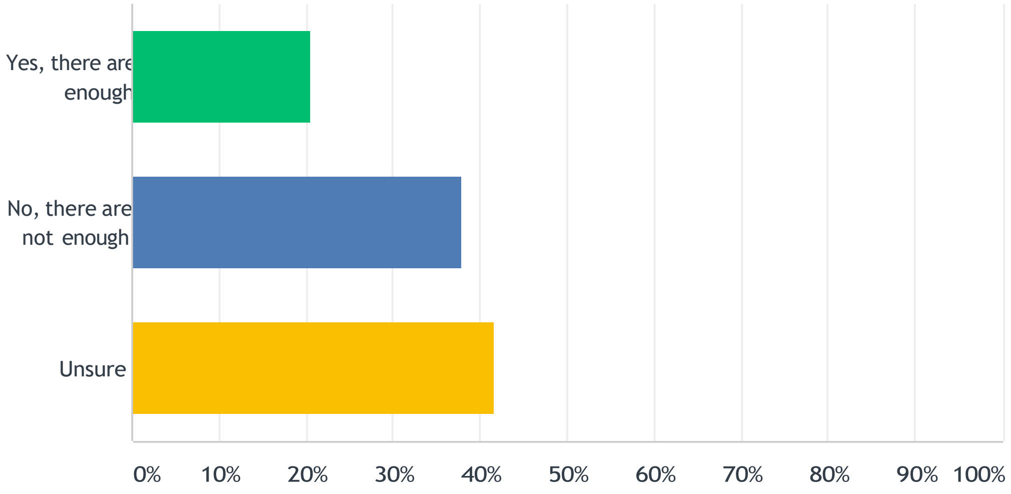


| ANSWER CHOICES | RESPONSES | |
| --- | --- | --- |
| Yes, there are enough | 20.50% | 90 |
| No, there are not enough | 37.81% | 166 |
| Unsure | 41.69% | 183 |
| TOTAL | | 439 |

# Supplementary Figure 28

## Compared to in-person education, how convenient was this course?

Answered: 439 Skipped: 0

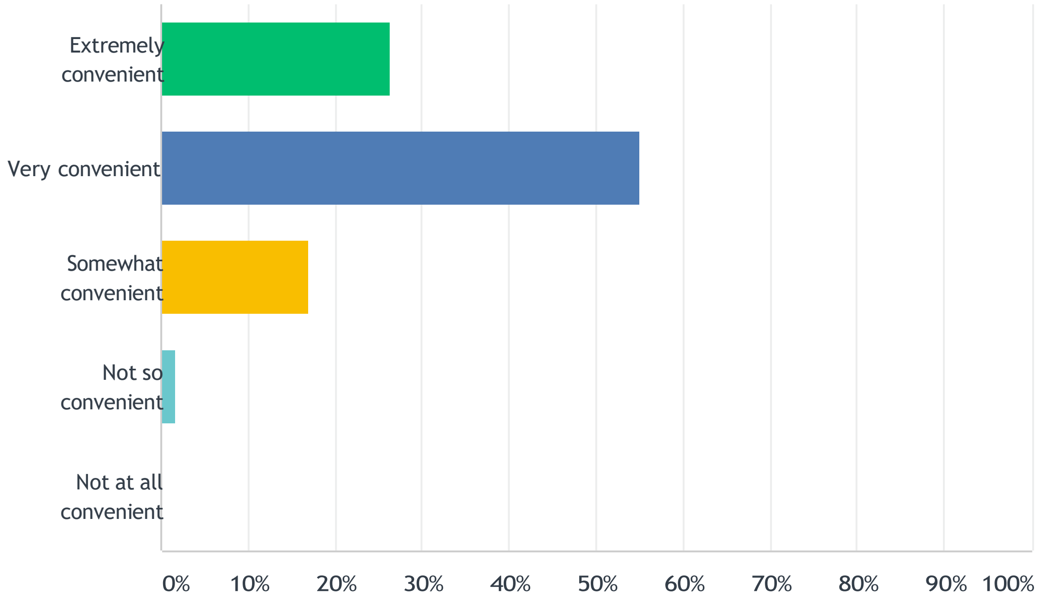


| ANSWER CHOICES | RESPONSES | |
|---|---|---|
| Extremely convenient | 26.42% | 116 |
| Very convenient | 55.13% | 242 |
| Somewhat convenient | 16.86% | 74 |
| Not so convenient | 1.59% | 7 |
| Not at all convenient | 0.00% | 0 |
| TOTAL | | 439 |

# Supplementary Figure 29

## Compared to in-person education, how effective was this course?

Answered: 439 Skipped: 0

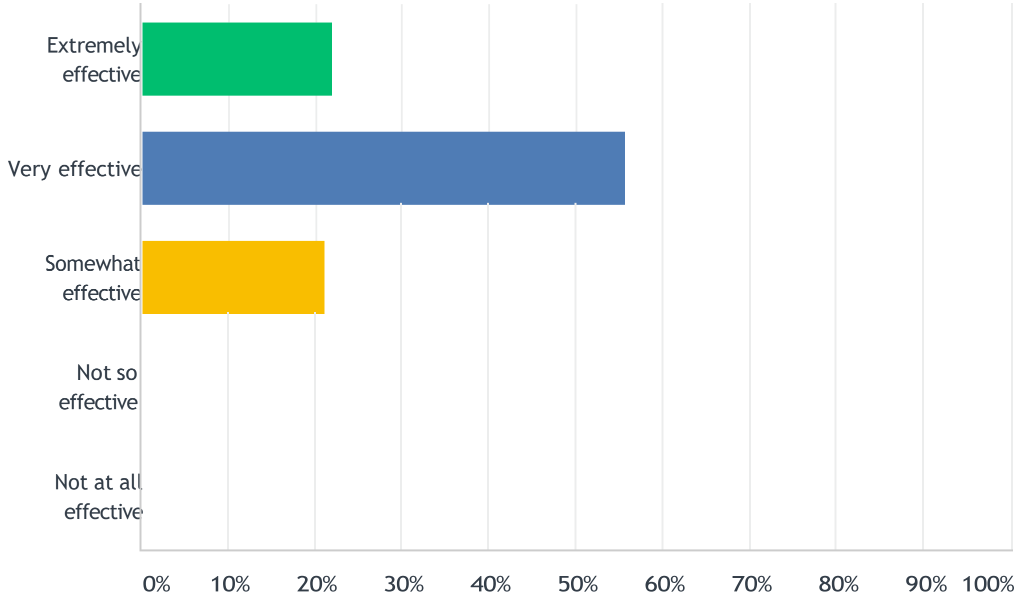


| ANSWER CHOICES | RESPONSES | |
|---|---|---|
| Extremely effective | 21.87% | 96 |
| Very effective | 55.58% | 244 |
| Somewhat effective | 21.18% | 93 |
| Not so effective | 1.37% | 6 |
| Not at all effective | 0.00% | 0 |
| TOTAL | | 439 |

# Supplementary Figure 30

## How likely are you to recommend this program to others?

Answered: 439 Skipped: 0

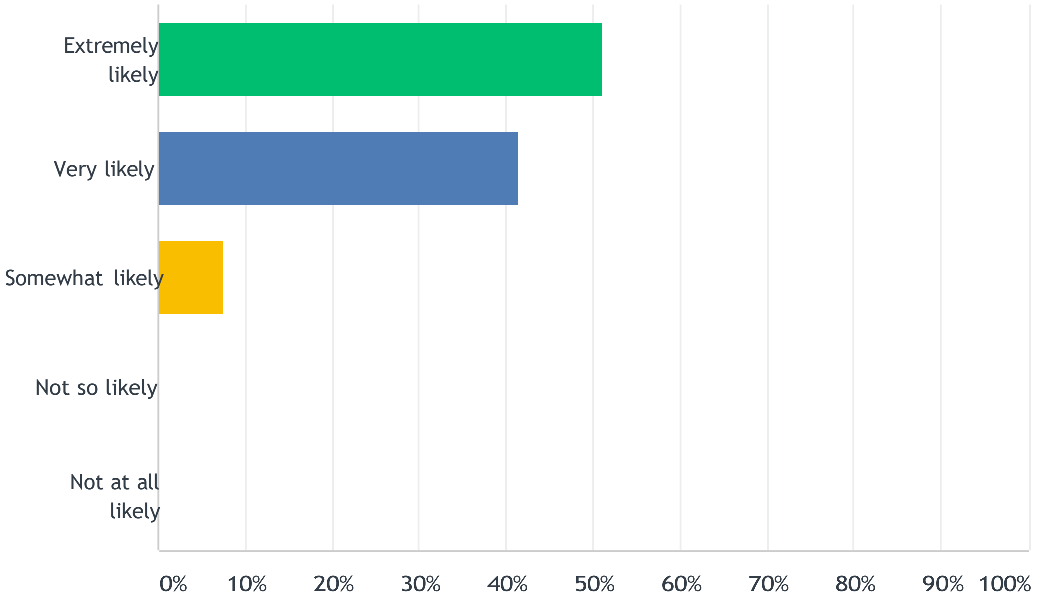


| ANSWER CHOICES | RESPONSES | |
|---|---|---|
| Extremely likely | 51.03% | 224 |
| Very likely | 41.46% | 182 |
| Somewhat likely | 7.52% | 33 |
| Not so likely | 0.00% | 0 |
| Not at all likely | 0.00% | 0 |
| TOTAL | | 439 |

# Supplementary Figure 31

## This course prepared me to understand more advanced topics in radiology

Answered: 439 Skipped: 0

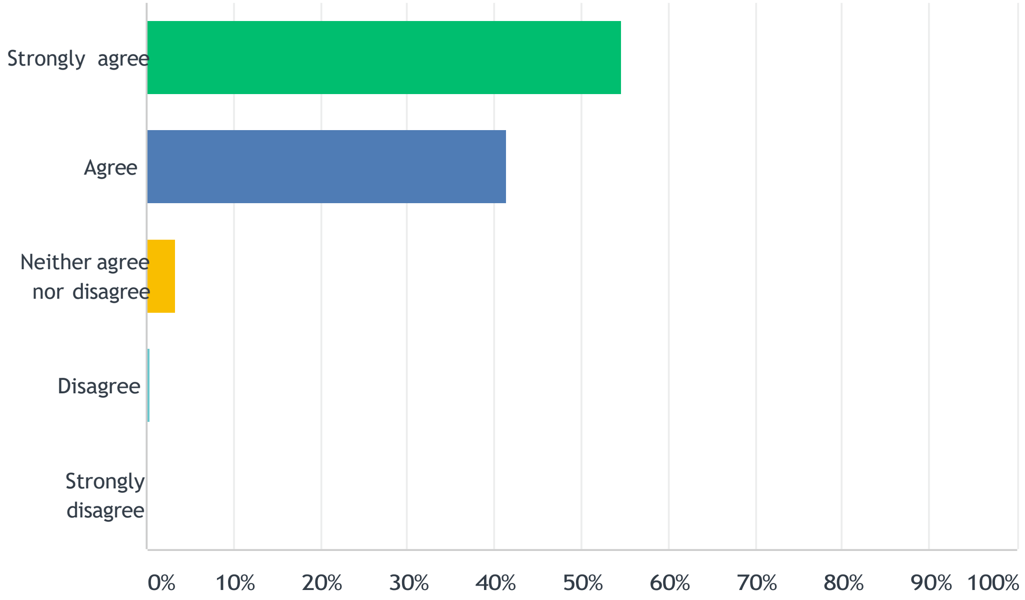


| ANSWER CHOICES | RESPONSES | |
|---|---|---|
| Strongly agree | 54.67% | 240 |
| Agree | 41.46% | 182 |
| Neither agree nor disagree | 3.42% | 15 |
| Disagree | 0.46% | 2 |
| Strongly disagree | 0.00% | 0 |
| TOTAL | | 439 |

# Supplementary Figure 32

## Introductory principles and concepts should be presented at the beginning of undergraduate radiology curricula

Answered: 439    Skipped: 0

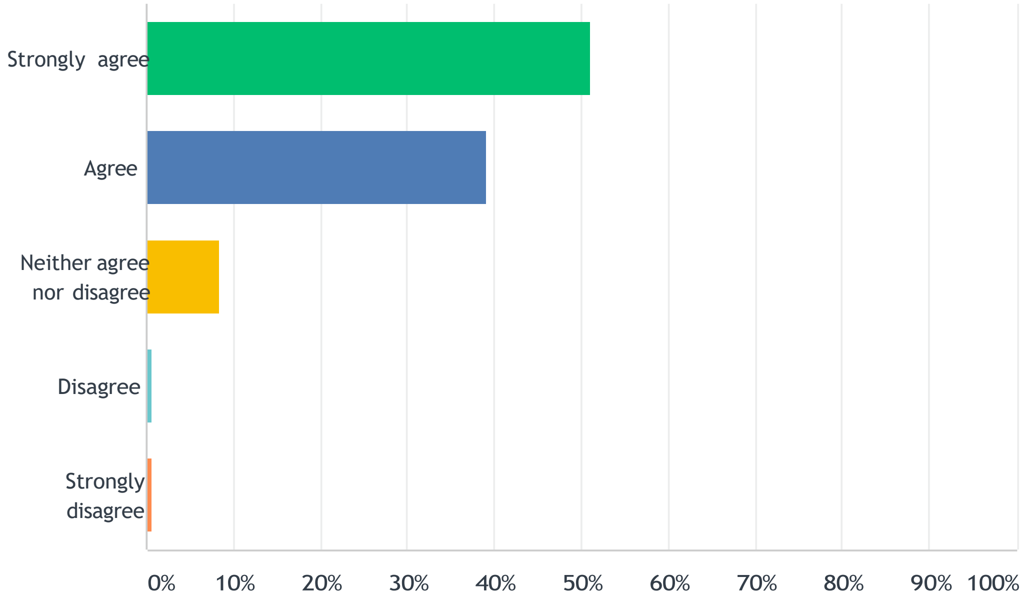


| ANSWER CHOICES | RESPONSES | |
|---|---|---|
| Strongly agree | 51.03% | 224 |
| Agree | 39.18% | 172 |
| Neither agree nor disagree | 8.43% | 37 |
| Disagree | 0.68% | 3 |
| Strongly disagree | 0.68% | 3 |
| TOTAL | | 439 |

# Supplementary Figure 33
## This course helped to bridge one or more of my radiology education gaps

Answered: 439 Skipped: 0

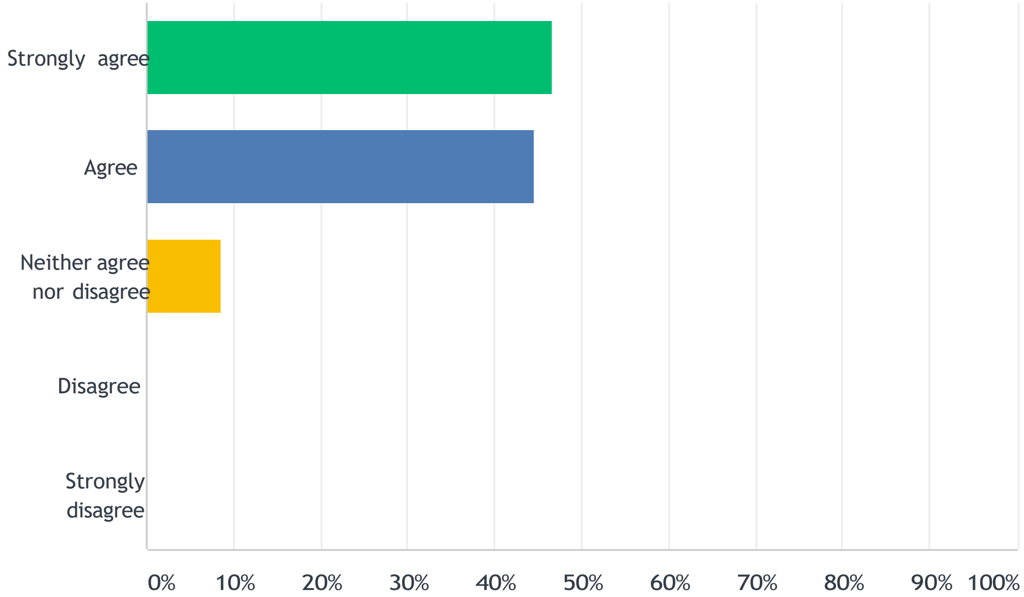


| ANSWER CHOICES | RESPONSES | |
|---|---|---|
| Strongly agree | 46.70% | 205 |
| Agree | 44.65% | 196 |
| Neither agree nor disagree | 8.66% | 38 |
| Disagree | 0.00% | 0 |
| Strongly disagree | 0.00% | 0 |
| TOTAL | | 439 |

# Supplementary Figure 34
# How did you hear about this course?

Answered: 439    Skipped: 0

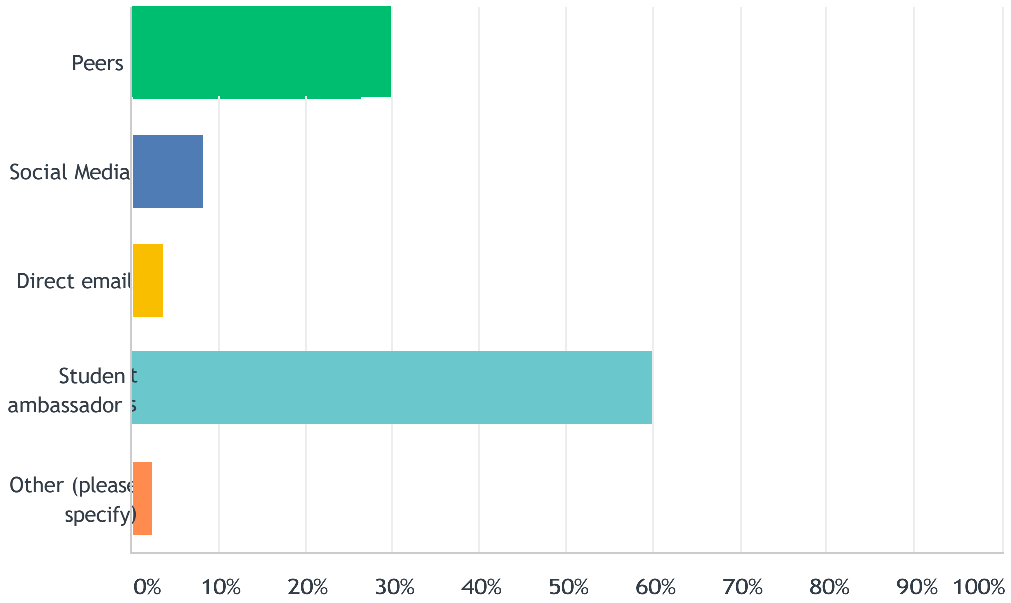


| ANSWER CHOICES | RESPONSES | |
|---|---|---|
| Peers | 26.42% | 116 |
| Social Media | 8.20% | 36 |
| Direct email | 3.64% | 16 |
| Student ambassadors | 59.45% | 261 |
| Other (please specify) | 2.28% | 10 |
| TOTAL | | 439 |

## Supplementary Figure 35

### Please provide suggestions on ways to improve this program's learning experience.

Answered: 136 Skipped: 303
